# Assessing the strength of evidence for a causal effect of respiratory syncytial virus lower respiratory tract infections on subsequent wheezing illness: a systematic review and meta-analysis

**DOI:** 10.1016/S2213-2600(20)30109-0

**Published:** 2020-08

**Authors:** Steven M Brunwasser, Brittney M Snyder, Amanda J Driscoll, Deshayne B Fell, David A Savitz, Daniel R Feikin, Becky Skidmore, Niranjan Bhat, Louis J Bont, William D Dupont, Pingsheng Wu, Tebeb Gebretsadik, Patrick G Holt, Heather J Zar, Justin R Ortiz, Tina V Hartert

**Affiliations:** a Department of Psychology, Rowan University, Glassboro, NJ, USA; b Vanderbilt University Medical Center, Nashville, TN, USA; c Centre for Vaccine Development and Global Health, School of Medicine, University of Maryland, Baltimore, MD, USA; d Children's Hospital of Eastern Ontario Research Institute, University of Ottawa, Ottawa, ON, Canada; e School of Public Health, Brown University, Providence, RI, USA; f Department of Immunizations, Vaccines and Biologicals, WHO, Geneva, Switzerland; g Independent Information Specialist, Ottawa, ON, Canada; h Center for Vaccine Innovation and Access, PATH, Washington, DC, USA; i Wilhelmina Children's Hospital and University Medical Centre Utrecht, Utrecht University, Utrecht, Netherlands; j Telethon Kids Institute, University of Western Australia, Perth, WA, Australia; j Red Cross War Memorial Children's Hospital and South African Medical Research Council Unit on Child and Adolescent Health, University of Cape Town, Cape Town, South Africa

## Abstract

**Background:**

Although a positive association has been established, it is unclear whether lower respiratory tract infections (LRTIs) with respiratory syncytial virus (RSV) cause chronic wheezing illnesses. If RSV-LRTI were causal, we would expect RSV-LRTI prevention to reduce the incidence of chronic wheezing illnesses in addition to reducing acute disease. We aimed to evaluate the strength of evidence for a causal effect of RSV-LRTI on subsequent chronic wheezing illness to inform public health expectations for RSV vaccines.

**Methods:**

We did a systematic review and meta-analysis of observational studies evaluating the association between RSV-LRTI and subsequent wheezing illness (exposure studies) and studies evaluating the association between RSV immunoprophylaxis and subsequent wheezing illness (immunoprophylaxis studies). Exposure studies were included if the exposure group members had an LRTI with laboratory-confirmed RSV and if the exposure ascertainment period began before 2 years of age and ended before 5 years of age. We required a wash-out period of more than 30 days between the index RSV-LRTI and the outcome measurement to allow for resolution of the acute illness. Comparisons between RSV-LRTI and non-RSV-LRTI were not included. Immunoprophylaxis studies were included if they measured the association with subsequent wheezing illness relative to a control group, either in a randomised controlled trial (RCT) or an observational design. For the immunoprophylaxis drugs in question, we required evidence of efficacy in targeting RSV-LRTI from at least one RCT to ensure biological plausibility. All variations of wheezing illness were combined into a single outcome that refers broadly to asthma or any other respiratory illness with wheezing symptoms. Ovid MEDLINE and Embase databases were searched from inception up to Aug 28, 2018. We evaluated whether data from exposure studies could provide evidence against the most viable non-causal theory that RSV-LRTI is a marker of respiratory illness susceptibility rather than a causal factor. Additionally, we tested whether RSV immunoprophylaxis reduces the odds of subsequent wheezing illnesses. We used a random-effects modelling framework and, to accommodate studies providing multiple correlated estimates, robust variance estimation meta-regressions. Meta-regression coefficients (*b*) quantify differences between exposure and comparator groups on the log_e_ odds ratio (log_e_ OR) scale.

**Findings:**

From 14 235 records we identified 57 eligible articles that described 42 studies and provided 153 effect estimates. 35 studies estimated the direct effect of RSV-LRTI on wheezing illnesses (exposure studies) and eight evaluated the effect of RSV immunoprophylaxis (immunoprophylaxis studies). Exposure studies that adjusted for genetic influences yielded a smaller mean adjusted OR estimate (aOR_+_ 2·45, 95% CI 1·23–4·88) compared with those that did not (4·17, 2·36–7·37), a significant difference (*b* 0·53, 95% CI 0·04–1·02). Infants who were not protected with RSV immunoprophylaxis tended to have higher odds of subsequent wheezing illness, as we would expect if RSV-LRTI were causal, but the effect was not significant (OR_+_ 1·21, 95% CI 0·73–1·99). There was generally a high threat of confounding bias in the observational studies. Additionally, in both the observational studies and immunoprophylaxis RCTs, there was high risk of bias due to missing outcome data.

**Interpretation:**

Our findings, limited to exposure and immunoprophylaxis studies, do not support basing policy decisions on an assumption that prevention of RSV-LRTI will reduce recurrent chronic wheezing illnesses.

**Funding:**

Bill & Melinda Gates Foundation.

## Introduction

Lower respiratory tract infections (LRTIs) caused by the respiratory syncytial virus (RSV) contribute substantially to infant (aged 0–1 years) morbidity and mortality,^1^, ^2^ making RSV prevention a global health priority.^3^ With policy makers committed to support the introduction of future licensed RSV vaccines,^3^, ^4^ there is a pressing need to estimate the full range of public health benefits. Although there is a well established positive association between RSV-LRTI and subsequent wheezing illness,^5^, ^6^, ^7^ it is unclear whether the association is causal.^8^ If the association were causal, efficacious RSV-LRTI prevention would probably reduce the burden of chronic wheezing illnesses in addition to acute disease, substantially increasing vaccine health benefits.^9^ In this Article, we evaluate the extent to which existing research supports a causal effect of RSV-LRTI on subsequent wheezing illness in young children. Although meta-analyses cannot resolve the question of causality, they can help appraise the strength of evidence for causality, which is critical for policy makers.

**Research in context**

**Evidence before this study**

Although there is a well established positive association between respiratory syncytial virus lower respiratory tract infections (RSV-LRTIs) and subsequent wheezing illnesses, it is unclear whether RSV-LRTI is a causal factor, a marker of susceptibility to respiratory illness, or both. Before doing the literature search for this Article, we searched PubMed and Google Scholar for articles published from inception until May 12, 2018. The full list of search terms is available in the appendix (pp 28–37). We identified two relevant meta-analytic reviews: the first reported a positive mean association (odds ratio [OR] 3·84) between RSV hospitalisation and subsequent asthma or wheezing in 15 studies; and the second also reported a significant mean association (OR 4·03) between RSV-bronchiolitis and asthma. In terms of causal evidence, a population-based birth cohort study showed that asthma risk was temporally associated with birth timing in close proximity to winter viral bronchiolitis epidemics, consistent with a causal effect. In contrast, twin-registry studies suggested that RSV-LRTI is more likely a marker of genetic risk than a causal contributor to asthma. One of the two randomised controlled trials of RSV immunoprophylaxis that evaluated effects on wheezing illness showed a reduction in parent-reported wheezing outcomes in those aged between 1 and 6 years, but neither trial found evidence of reductions in medically-attended wheezing illness.

**Added value of this study**

Although previous meta-analyses provided estimates of the association between RSV-LRTI and recurring wheezing illness, this Article was, to our knowledge, unique in that it had the explicit goal of appraising the strength of evidence for a causal effect. We could have increased confidence in a causal effect by providing evidence against the most plausible non-causal theory (ie, that RSV-LRTI is a marker of shared susceptibility to lung disease) and showing that RSV immunoprophylaxis protects against childhood wheezing illness. However, our findings did not support either of these conditions. These findings do not rule out a causal effect but suggest that the current evidence does not support basing policy decisions on an assumption that prevention of RSV-LRTI will reduce recurrent wheezing illnesses.

**Implications of all the available evidence**

Our findings, in combination with previous RSV twin studies, are consistent with the hypothesis that shared genetic predisposition accounts for a substantial proportion of the association between RSV-LRTI and subsequent wheezing. Individual studies and meta-analyses that do not account for shared genetic risk could substantially overestimate the effect of RSV-LRTI on recurring wheezing illness. Additionally, we found insufficient evidence that RSV immunoprophylaxis prevents wheezing illness, which we would expect if RSV-LRTI were causal. Long-term follow-up data from ongoing trials are needed before assuming RSV-LRTI prevention might lead to reduction in recurrent wheeze or asthma. Additionally, more research is needed to assess the potential effects of RSV infection without LRTI and potential host–viral interactions.

**Figure 1:**
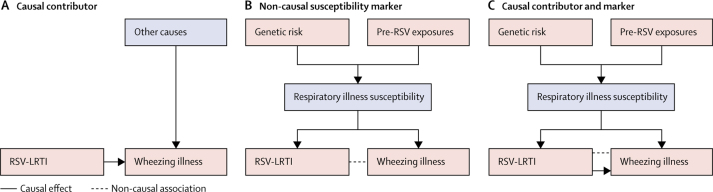
Three potential models to explain the observed association between RSV-LRTI and subsequent wheezing illness (A) RSV-LRTI as one of multiple causal contributors to wheezing illness. There is a directed solid arrow (representing a causal effect) connecting RSV-LRTI and wheezing illness. (B) A non-causal model in which the positive association between RSV-LRTI and subsequent wheezing illness is confounded by a pre-existing susceptibility to respiratory illnesses. According to this model, this pre-existing susceptibility is driven by genetics and early environmental insults that precede RSV-LRTI. The dotted, non-directional line connecting RSV-LRTI and wheezing illness represents a non-causal association. (C) The association between RSV-LRTI and subsequent wheezing illness is due partly to the confounding influence of pre-existing respiratory illness susceptibility and partly to a causal effect of RSV-LRTI. RSV-LRTI=respiratory syncytial virus lower respiratory tract infections.

We present three possible models for the established association between RSV-LRTI and wheezing illness (figure 1).^10^, ^11^ In the first model, RSV-LRTI is one of several causal contributors to subsequent wheezing illness (figure 1A). The potential mechanisms by which RSV infection could contribute to recurrent wheezing illness (eg, by degrading airway epithelial barriers and altering functioning of regulatory T cells) have been reviewed in other papers.^11^ The second is a non-causal model, in which a pre-existing susceptibility to respiratory illness causes both the RSV-LRTI and subsequent wheezing illness (ie, confounding; figure 1B).^10^, ^12^, ^13^ In this model, the pre-existing respiratory illness susceptibility is attributable to heritable factors and early environmental insults. Past research has shown that infants who develop RSV-LRTI have poorer pre-existing lung function^14^ (a highly heritable trait),^15^ which could make them susceptible to both severe illness in response to RSV infection (ie, RSV-LRTI) and recurrent wheezing illness.^12^, ^13^, ^16^ In the third and final model, the association is attributable partly to a causal effect and partly to the confounding influence of pre-existing respiratory illness susceptibility (figure 1C).^11^

In this Article, we use meta-regression to consider both causal and non-causal models of the association between RSV-LRTI and subsequent wheezing illness and evaluate whether their unique implications (ie, what must be true if the models were correct) are consistent with empirical data.^17^ The non-causal model in figure 1B has testable implications^17^ for studies evaluating the association between early life RSV-LRTI and subsequent wheezing illness (exposure studies): studies adjusting for contributors to, or markers of, pre-existing respiratory susceptibility should yield smaller effect estimates.^18^ If they do not, this would provide evidence against the non-causal explanation, increasing the plausibility of a causal effect.^19^

Studies evaluating the association between RSV immunoprophylaxis and subsequent wheezing illness (immunoprophylaxis studies) can also help to appraise the strength of evidence for a causal effect (figure 1A). If RSV-LRTI were a causal factor, then efficacious immunoprophylaxis would most likely reduce the risk of wheezing illness. Meta-analysis could therefore be used to test whether RSV immunoprophylaxis is associated with a decreased risk of subsequent wheezing illness.^18^ In standard care, infants receiving RSV immunoprophylaxis tend to have poorer baseline health status than those who do not, even within groups at high risk.^20^, ^21^ Consequently, observational RSV immunoprophylaxis studies require particular care to limit confounding by indication,^22^ in addition to other biases.^18^ Properly done immunoprophylaxis randomised controlled trials (RCTs) eliminate confounding by indication.^18^, ^23^ However, drawing causal inferences about the effect of RSV-LRTI on wheezing illness in these RCTs requires the assumption that treatment assignment (eg, immunoprophylaxis *vs* placebo) affects risk for wheezing illness only by limiting the severity of RSV-LRTI exposure and not through any other mechanisms.^24^, ^25^

We were commissioned by WHO to do a systematic review and meta-analysis to assess the evidence for a causal effect of RSV-LRTI on subsequent wheezing illness to inform public health expectations for RSV vaccines.

## Methods

### Search strategy and selection criteria

The protocol for this systematic review and meta-analysis provides complete methodological details (appendix pp 3–27). As there was no involvement of human participants or identifiable information, institutional review board approval was not required. We have described the two types of published peer-reviewed studies included in the analysis and all the inclusion criteria (panel, table 1). RSV-LRTI exposure studies measured the direct association between RSV-LRTI exposure and subsequent wheezing illness. Immunoprophylaxis studies measured the association between RSV immunoprophylaxis and subsequent wheezing illness.

Panel **Research designs of relevant studies**

**Respiratory syncytial virus (RSV)-associated lower respiratory tract infection (LRTI) exposure studies**

Evaluate the direct association between RSV-LRTI and subsequent wheezing illness using an observational design

*Viral surveillance studies*

Do viral surveillance over a discrete exposure ascertainment period: those developing RSV-LRTI during this period form the exposure group and those with no LRTI form the comparison group

*Medical event studies*

Compare children (aged 0–5 years) with an RSV-LRTI medical event (eg, hospital admission, acute medical visit, etc) during the exposure ascertainment period to those without an LRTI-related medical event (comparator group)

**RSV immunoprophylaxis studies**

Evaluate the association between receipt of RSV immunoprophylaxis with established efficacy and subsequent wheezing illness

*Randomised controlled trial*

Randomly assign infants (aged 0–1 years) to receive either RSV immunoprophylaxis or placebo

*Observational (non-randomised) study*

Compare infants (aged 0–1 year) receiving RSV immunoprophylaxis in their normal clinical care to those not receiving immunoprophylaxis (ie, intervention not determined by study procedures)

**Table 1:** Summary of inclusion and exclusion criteria and PICOS literature search framework

	**RSV-LRTI exposure studies**	**RSV immunoprophylaxis studies**
Population characteristics	Human participants	Human participants
Intervention or exposure*	RSV-LRTI during a period beginning <2 years of age and fully contained within ages 0–5 years (operationalised as an exposure or mediator variable)	RSV immunoprophylaxis with established efficacy (either from the trial in question or past RCTs) in preventing or mitigating RSV-LRTI
Comparator	LRTI absent or undetected during the exposure period	RSV immunoprophylaxis not received during the exposure period
Outcome	Wheezing illness measured subsequent† to the index RSV-LRTI illness that defines inclusion in the exposure *vs* comparator groups	Wheezing illness subsequent to study intervention protection period
Study design	Study analysed quantitative data and was published (including online only publication ahead of print) in English language in a peer-reviewed journal before the final search date; exposure and comparator groups sampled from the same population; method of ascertaining exposure and outcomes were the same for exposure and comparator groups	As for exposure studies

PICOS=population, intervention or exposure, comparator, outcome, study design. RSV=respiratory syncytial virus. LRTI=lower respiratory tract infection. RCT=randomised controlled trial.

* Clinical trials might estimate the effect of RSV-LRTI on asthma or wheezing outcomes indirectly by reporting the effect of immunoprophylaxis on asthma or wheezing outcomes and report the direct association between RSV-LRTI and asthma or wheezing outcomes.

† Defined as occurring >30 days after the index RSV-LRTI.

RSV-LRTI exposure studies were included if the exposure group members had an LRTI with laboratory-confirmed RSV. LRTI was considered present if children were diagnosed with relevant illnesses (eg, bronchiolitis or pneumonia), had relevant clinical indications (eg, wheeze), or received treatment in hospital for RSV-related illnesses. As we were interested in early life RSV-LRTI, the exposure ascertainment period had to begin before 2 years of age and end before 5 years. These exposure studies could be further categorised by study design. Some exposure studies determined RSV-LRTI status by doing viral surveillance over a defined ascertainment period (surveillance studies), whereas others compared individuals with an RSV-LRTI medical event (eg, treatment in hospital) to those without an LRTI medical event (medical event studies). We required a wash-out period of more than 30 days^26^ between the index RSV-LRTI and the outcome measurement to allow for resolution of the acute illness. Comparisons between RSV-LRTI and non-RSV-LRTI were not included because they do not directly address the causal question of interest, which pertains to RSV-LRTI in particular and not LRTI more generally.

We included RSV immunoprophylaxis studies if they measured the association with subsequent wheezing illness relative to a control group, either in an RCT or an observational design. For the immunoprophylaxis drugs in question, we required evidence of efficacy in targeting RSV-LRTI from at least one RCT^27^, ^28^ to ensure biological plausibility. Because studies did not use consistent definitions for asthma and other wheezing illnesses (eg, what some authors termed asthma, others labelled recurrent wheeze), all variations of wheezing illness were combined into a single outcome. Wheezing illness, therefore, refers broadly to asthma or any other respiratory illness with wheezing symptoms. To evaluate whether associations were driven by transient wheezing illnesses, we did a sensitivity analysis including only outcomes described as asthma measured at 6 years or older, when asthma can be diagnosed reliably.^29^

An expert systematic review information specialist did a literature search of Ovid MEDLINE and Embase databases using the population, intervention or exposure, comparator, outcome, study design framework,^30^ in English (table 1). The original search strategy was reviewed by an independent information specialist using the Peer Review of Electronic Search Strategies checklist,^31^ evaluated against a test set of scholarly articles with known relevance, and subsequently refined to ensure comprehensiveness. The final search was done on Aug 28, 2018, with no restriction on publication dates, and the search terms can be found in the appendix (pp 28–37).

Four study investigators (AJD, BMS, JRO, and SMB) with doctoral degrees in health-care sciences completed record reviews and data abstraction. Literature search records were reviewed in two stages. Stage 1 eliminated records that were irrelevant based on the study abstracts alone. Two of these investigators evaluated each abstract independently, blinded to the others' ratings. Any article deemed potentially relevant by either reviewer was retained. In stage 2, two investigators independently and redundantly reviewed the full text of the articles that passed stage 1 to determine whether the full inclusion criteria were met.

For each article, one of the four investigators abstracted relevant data and a different investigator did quality assurance reviews, resolving discrepancies by consensus. For estimates from RSV-LRTI exposure studies, we noted whether or not analyses controlled for genetic predisposition to wheezing illness and neonatal health proxies that might be markers of respiratory illness predisposition (eg, preterm birth). As RSV often co-occurs with other respiratory infections that are also positively associated with subsequent wheezing illness,^32^ it is plausible that the effect of these co-infections could be misattributed to RSV. Therefore, we also coded whether studies adjusted for non-RSV viral or bacterial respiratory co-infections. Additionally, we extracted data on the following key study features (table 2): country income level, asthma risk-based versus non-risk-based enrolment, age at outcome ascertainment (preschool 0–4 years; school 5–12 years; adolescence 13–18 years; or adulthood ≥19 years old), and timing of exposure ascertainment (limited to the first year of life or not). Finally, we coded whether immunoprophylaxis studies were RCTs or observational.

**Table 2:** Study characteristics

		**Countries**	Country income level*	**Exposure ascertainment**†	**Estimates included**‡	**Measured asthma (age ≥6 years)**§	**Outcomes age groups**¶	**Enrolment strategy**‖
**RSV immunoprophylaxis studies**								
Randomised controlled trials								
	Blanken et al (2013);^33^ Scheltema et al (2018)^34^	Netherlands	High	Limited to first year of life	6	Yes	Preschool and primary school	Risk based: preterm birth
	O'Brien et al (2015)^35^	USA	High	Limited to first year of life	3	No	Preschool	Risk based: ethnic groups at high risk
Observational studies								
	Carroll et al (2017)^20^	USA	High	Limited to first year of life	3	No	Primary school	Risk based: chronic lung disease or preterm birth
	Yoshihara et al (2013);^36^ Mochizuki et al (2017)^37^	Japan	High	Limited to first year of life	3	Yes	Preschool	Risk based: preterm birth
	Simoes et al (2007)^38^	Spain, Germany, Netherlands, Canada, Poland, and Sweden	High	Limited to first year of life	2	No	Preschool	Risk based: preterm birth
	Prais et al (2016)^39^	Israel	High	Limited to first year of life	3	No	Preschool	Risk based: preterm birth
	Haerskjold et al (2017)^21^	Denmark and Sweden	High	Extends beyond first year of life	4	No	Preschool	Not risk based
	dos Santos Simões et al (2019)^40^	Brazil	Upper-middle	Extends beyond first year of life	1	No	Preschool	Risk based: preterm birth and referred for RSV immunoprophylaxis
**RSV-LRTI exposure studies**								
Medical event studies								
	Ruotsalainen et al (2013);^41^ Backman et al (2018)^42^	Finland	High	Extends beyond first year of life	3	Yes	Adolescence	Not risk based
	Korppi et al (1994);^43^ Korppi et al (2004);^44^ Ruotsalainen et al (2010);^45^, ^46^ Backman et al (2014)^47^	Finland	High	Extends beyond first year of life	11	Yes	Primary school, adolescence, and adulthood	Not risk based
	Sigurs et al (1995, 2000, 2005, 2010)^48^, ^49^, ^50^, ^51^	Sweden	High	Limited to first year of life	10	Yes	Preschool, primary school, and adolescence	Not risk based
	Poorisrisak et al (2010)^16^	Denmark	High	Extends beyond first year of life	1	Yes	Primary school	Not risk based
	Fjaerli et al (2005)^52^	Norway	High	Limited to first year of life	2	Yes	Primary school	Not risk based
	Henderson et al (2005)^53^	UK	High	Limited to first year of life	3	Yes	Preschool and primary school	Not risk based
	Stensballe et al (2018)^54^	Denmark	High	Extends beyond first year of life	2	No	Preschool	Not risk based
	Carbonell-Estrany et al (2015)^55^	Spain	High	Limited to first year of life	3	No	Preschool	Risk based: preterm birth
	Escobar et al (2013)^56^	USA	High	Limited to first year of life	12	No	Preschool	Not risk based
	Kim et al (2013)^57^	South Korea	High	Extends beyond first year of life	1	No	Preschool	Not risk based
	Palmer et al (2011)^58^	USA	High	Limited to first year of life	4	No	Preschool	Risk based: preterm birth
	Blanken et al (2016)^59^	Netherlands	High	Limited to first year of life	1	No	Preschool	Risk based: preterm birth
	Bloemers et al (2010)^60^	Netherlands	High	Extends beyond first year of life	3	No	Preschool	Risk based: Down syndrome
	García-García et al (2007)^61^	Spain	High	Extends beyond first year of life	3	No	Preschool	Not risk based
	Mikalsen et al (2012)^62^	Norway	High	Limited to first year of life	1	Yes	Primary school	Not risk based
	Osundwa et al (1993)^63^	Qatar	High	Limited to first year of life	1	No	Preschool	Not risk based
	Palmer et al (2010)^64^	USA	High	Limited to first year of life	3	No	Preschool	Not risk based
	Weber et al (1999)^65^	The Gambia	Low	Limited to first year of life	1	No	Preschool	Not risk based
	Fauroux et al (2014)^66^	France	High	Limited to first year of life	2	No	Preschool	Risk based: preterm birth
	Sims et al (1978)^67^	UK	High	Limited to first year of life	1	No	Primary school	Not risk based
	Pullan & Hey (1982)^68^	UK	High	Limited to first year of life	1	No	Primary school	Not risk based
	Juntti et al (2003)^69^	Finland	High	Limited to first year of life	3	Yes	Primary school	Not risk based
	Bertrand et al (2015)^70^	Chile	High	Limited to first year of life	2	No	Preschool	Not risk based
	Singleton et al (2003)^71^	USA	High	Extends beyond first year of life	4	No	Preschool and primary school	Risk based: ethnic group at high risk
	Schauer et al (2002)^72^	Germany	High	Limited to first year of life	2	No	Preschool	Not risk based
	Stensballe et al (2009)^73^	Denmark	High	Extends beyond first year of life	7	No	Preschool	Not risk based
	Munywoki et al (2013)^74^	Kenya	Lower-middle	Limited to first year of life	1	No	Preschool	Not risk based
Viral surveillance studies								
	Kusel et al (2007, 2012)^32^, ^75^	Australia	High	Limited to first year of life	10	Yes	Preschool and primary school	Risk based: family history of asthma or atopy
	Calişkan et al (2013);^76^ Bønnelykke et al (2015)^77^	Denmark	High	Extends beyond first year of life	3	Yes	Primary school	Risk based: family history of asthma or atopy
	Calişkan et al (2013);^76^ Lemanske et al (2005);^78^ Jackson et al (2008);^79^ Rubner et al (2017)^80^	USA	High	Extends beyond first year of life	11	Yes	Preschool or primary school	Risk based: family history of asthma or atopy
	Stein et al (1999);^81^ Voraphani et al (2014)^82^	USA	High	Extends beyond first year of life	10	Yes	Primary school and adulthood	Not risk based
	Drysdale et al (2015)^83^	UK	High	Limited to first year of life	2	No	Preschool	Risk based: preterm birth
	Zomer-Kooijker et al (2014)^84^	Netherlands	High	Limited to first year of life	3	Yes	Primary school	Not risk based
	Broughton et al (2007)^85^	UK	High	Limited to first year of life	1	No	Preschool	Risk based: preterm birth

RSV=respiratory syncytial virus. LRTI=lower respiratory tract infection.

* Determined using the World Bank classifications.^86^

† Was the exposure ascertainment period limited to the first year of life or did it extend beyond?

‡ Number of effect size estimates that contributed to the primary analysis.

§ At least one outcome was described as asthma and measured at ≥6 years of age.

¶ Age category when outcomes measured: preschool 0–4 years; primary school 5–12 years; adolescence 13–18 years; and adulthood ≥19 years.

‖ Was enrolment limited to individuals with known risk for wheezing illness other than early life LRTI?

### Data analysis

We quantified differences between exposure and comparator groups using the log_e_ odds ratio (log_e_OR). Whenever possible, for RSV-LRTI exposure studies and non-randomised immunoprophylaxis studies, we included estimates from analyses in which there was explicit effort to adjust for confounders, as these estimates were generally expected to be less biased than unadjusted estimates. Some studies reported other adjusted ratio-based effect estimates (eg, adjusted risk ratios). Rather than excluding these estimates or replacing them with unadjusted ORs, we included them in our analyses as the best-available estimates. 17% of estimates (26 of 153) used non-OR ratio estimates as stand ins for OR estimates, which pulls the estimated mean OR systematically toward the null.^87^ We used unadjusted estimates for the effect of RSV immunoprophylaxis on wheezing illness in RCTs. Most studies provided multiple correlated estimates. To make optimal use of the available data,^88^ we included all relevant estimates that provided at least some unique information.

To accommodate studies providing multiple correlated estimates, we did robust variance estimation (RVE) meta-regressions^89^ using robumeta version 2.0^90^ in R version 3.5.0. RVE allows studies to contribute multiple correlated estimates while providing accurate point estimates and confidence intervals.^88^, ^89^ For exposure studies, we regressed effect estimates on study-level covariates selected a priori to test study hypotheses and account for variability in effect estimates across studies. Meta-regression coefficient estimates (*b*) are reported to quantify covariate effects on the log_e_OR scale.

We used the Newcastle-Ottawa Scale^91^ for observational studies and the Cochrane Risk of Bias Tool^92^ for RCTs, to identify potential biases in the extant literature and corresponding priorities for improving future study design.

### Role of the funding source

This study was commissioned by WHO through a grant from the Bill & Melinda Gates Foundation. A WHO RSV expert (DRF) contributed to the study conceptual isation, protocol and search strategy development, interpretation of results, and manuscript editing. AJD was supported by the National Institute of Health (NIH). The NIH had no role in the study design, data collection, analysis, interpretation of data, writing of the report, or in the decision to submit the paper for publication. The Gates Foundation had no role in study design, data collection, data analysis, data interpretation, or writing of the report. The corresponding author had full access to all the data in the study and had final responsibility for the decision to submit for publication.

## Results

**Figure 2:**
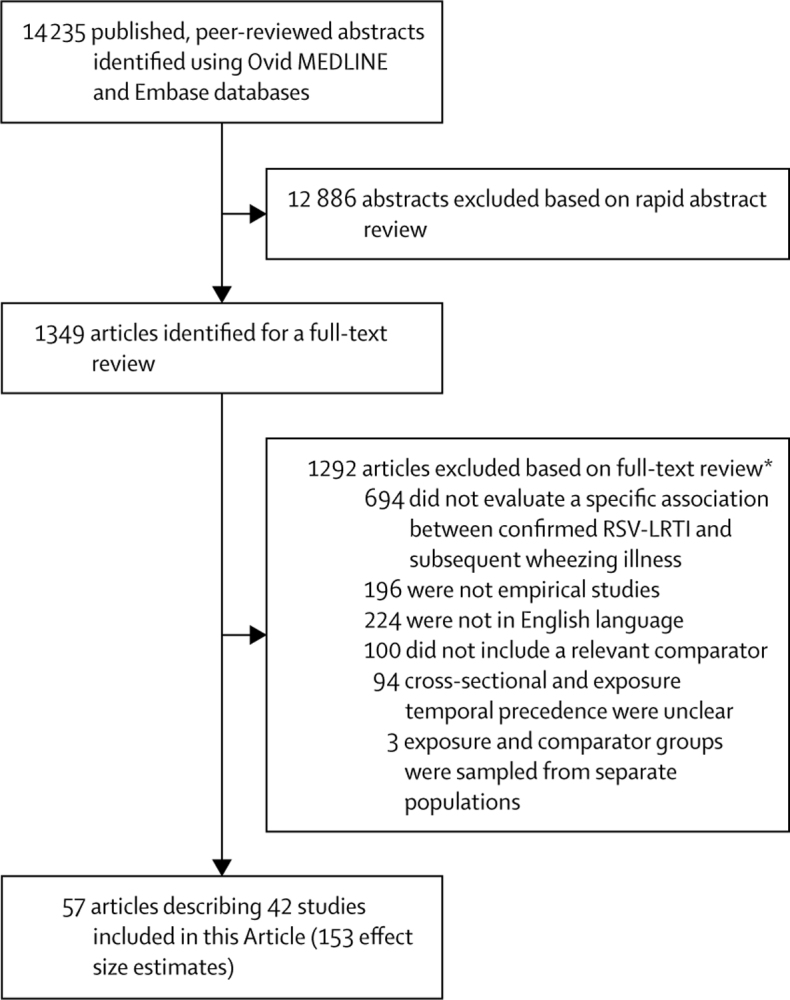
Study selection RSV-LRTI=respiratory syncytial virus lower respiratory tract infections. *Reviewers sometimes selected multiple inclusion criteria that were not met; hence, the numbers associated with specific reasons for exclusion do not add up to the total number of articles excluded.

From 14 235 initial records, we identified 57 eligible articles, describing 42 studies, and providing 153 effect estimates (table 2 and figure 2). In addition to the 34 RSV-LRTI exposure studies (table 2), one of the immunoprophylaxis studies (Scheltema et al)^34^ provided an estimate of the direct effect of RSV-LRTI on wheezing illnesses. Therefore, 35 studies estimated the direct effect of RSV-LRTI on wheezing illnesses and eight studies evaluated the effect of immunoprophylaxis on wheezing illnesses. All but two studies were done in high-income countries (table 2). A common potential source of bias, among both RSV-LRTI exposure studies and immunoprophylaxis studies, was that missing outcome data were treated as completely random without clear justification (appendix pp 40–48).^93^ It is unclear whether the suboptimal treatment of missing data is more likely to bias towards a larger or smaller estimate of the effect of RSV-LRTI on subsequent wheezing illness. Additionally, it was often impossible to know with certainty, particularly in medical event studies, whether RSV-LRTI preceded early wheezing illness manifestations that might have gone undetected. This would probably result in inflated effect estimates as the exposure cannot follow the outcome it purportedly causes.^94^

Among RSV-LRTI exposure studies, the unconditional mean OR (OR_+_) indicated that children exposed to RSV-LRTI had a 3·39 times increase in odds of subsequent wheezing illness (95% CI 2·72–4·24). OR_+_ remained positive when limiting the analysis to effect estimates of RSV-LRTI on asthma outcomes measured at age 6 years and older (41 estimates from 14 studies, OR_+_ 2·64, 95% CI 1·75–3·98).

**Figure 3:**
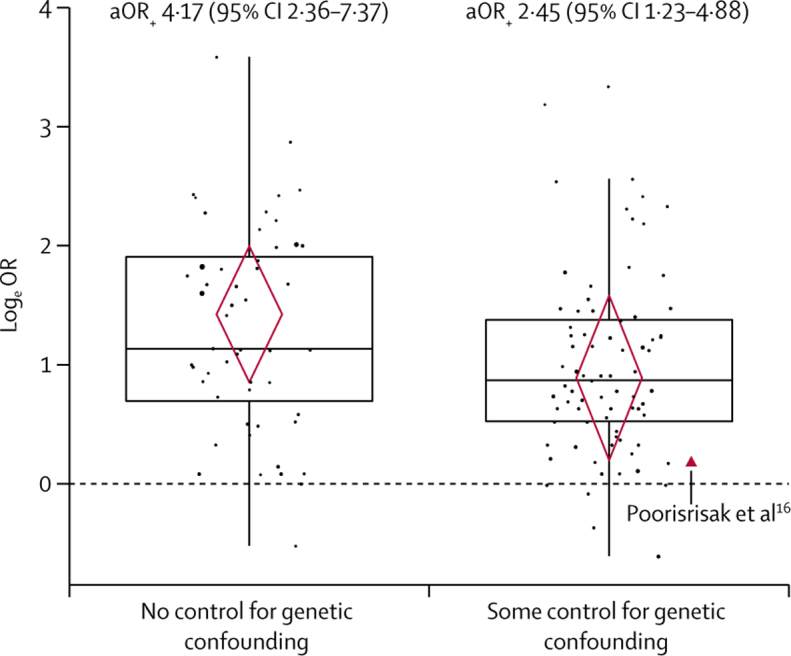
Observed effect size distributions and conditional mean effect sizes for studies that did and did not control for genetic confounding Effect estimates from respiratory syncytial virus lower respiratory tract infections exposure studies controlling for potential genetic influences (n=77) were smaller, on average, than those that did not control for potential genetic influences (n=52). The y-axis represents log_e_OR estimates, with 0 indicating no effect (dashed line). Points represent observed individual effect estimates and their size is proportional to their inverse-variance weights (ie, more precise estimates have larger points). The estimate provided by Poorisrisak and colleagues^16^ is displayed as a red triangle and annotated as it is the only estimate in which genetic influences were eliminated completely. The boxplots display characteristics of the distributions of the observed effect estimates (eg, medians and IQRs). The centres of the red diamonds represent the conditional mean log_e_OR estimates based on the primary meta-regression model. The bottom and top points of the diamonds represent, respectively, the lower and upper bounds of the 95% CIs. Mean aOR_+_ and 95% Cls based on the primary meta-regression model are provided for each group. Log_e_OR=log_e_ odds ratio. aOR_+_=adjusted OR estimates.

In our primary model, effect estimates for the association between RSV-LRTI and wheezing illness differed depending on whether estimates were adjusted for genetic influences (*b* 0·53, 95% CI 0·04–1·02). As shown in figure 3, the adjusted mean OR (aOR_+_) was considerably smaller when estimates accounted for genetic influences (n=77, aOR_+_ 2·45, 95% CI 1·23–4·88) relative to those that did not (n=52, aOR_+_ 4·17, 2·36–7·37). The one study^16^ that eliminated genetic influences by comparing wheezing illness outcomes in monozygotic twins with discordant RSV-LRTI status had a point estimate (OR 1·21) smaller than 88% (68 of 77) of estimates in which genetic influences were only partly controlled. However, uncertainty was high in this study with 95% CI values ranging from highly protective to highly damaging (0·36–4·00).^16^ There was no evidence that effect estimates varied depending on whether they controlled for differences in neonatal health or co-infections. The effect of controlling for genetic influences was robust when simultaneously removing all unadjusted estimates (*b* 0·63, 95% CI 0·05–1·20). However, when sequentially removing one study at a time (and all its estimates) from the analysis, eight studies^32^, ^54^, ^58^, ^59^, ^64^, ^69^, ^73^, ^85^ had enough influence that their removal nullified the effect of controlling for genetic influences (appendix p 49).

**Figure 4:**
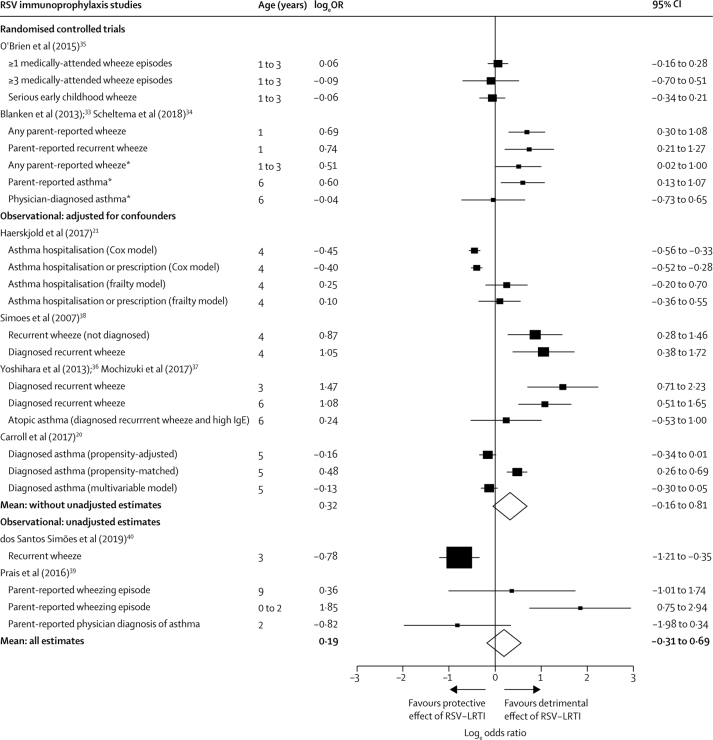
Forest plot evaluating whether infants who did not receive RSV immunoprophylaxis had increased odds of subsequent wheezing illness If RSV-LRTI were a cause of subsequent wheezing illness, then we would expect infants (aged 0-1 years) not receiving RSV immunoprophylaxis to have greater odds of developing subsequent wheezing illness compared with infants at similar risk who do receive RSV immunoprophylaxis. Our analyses from RSV immunoprophylaxis studies provided insufficient evidence for this hypothesis. In the figure, log_e_OR >0 indicate greater odds of subsequent wheezing illness in children who did not receive immunoprophylaxis. The higher of the two diamonds depicts the weighted mean log_e_OR for randomised trials and observational studies that adjusted for confounders. The lower of the two diamonds depicts the weighted mean log_e_OR across all estimates, including those from observational studies that did not adjust for confounders. Neither mean estimate was significantly greater than 0. RSV-LRTI=respiratory syncytial virus lower respiratory tract infection. Log_e_OR= log_e_ odds ratios. *Estimates from the Blanken et al^33^ and Scheltema et al^34^ were based on outcomes measured after the blinding of study participants had been broken at 1 year of age, although assessors were blinded throughout the study.

In our primary model of RSV immunoprophylaxis studies, the mean effect estimate was positive (OR_+_ 1·21), indicating that those not receiving immunoprophylaxis (with presumably greater risk of RSV-LRTI) tended to have higher odds of subsequent wheezing illness, but the 95% CI (0·73–1·99) included the null (figure 4). The mean effect size was slightly larger but remained non-statistically significant when removing two studies^39^, ^40^ that did not adjust for confounders (OR_+_ 1·38, 95% CI 0·85–2·24). Owing to the small sample size (eight estimates), there was considerable uncertainty around the mean estimate among the two RCTs and no evidence of increased odds of wheezing illness among children who did not receive RSV immunoprophylaxis (OR_+_ 1·24, 0·04–36·27).

## Discussion

Although we cannot rule out a causal effect of RSV-LRTI on subsequent wheezing illness, neither of our two primary findings support the case for causality. First, RSV-LRTI exposure studies controlling for genetic influences produced smaller effect estimates, consistent with what we would expect if RSV-LRTI were at least partly a marker of genetic susceptibility rather than a purely causal association. Although this finding was not fully robust in a leave-one-out sensitivity analysis, all but one^16^ of the studies controlling for genetic influences measured genetic risk using imperfect proxies (eg, familial asthma history) and could only partly remove heritable influences. Consequently, our models probably underestimated the influence of adjusting for genetics.^95^, ^96^ In sum, the mean effect estimate for exposure studies was positive and significant even among studies that adjusted for proxies of genetic risk; however, it is possible that some or much of the effect is attributable to residual heritable influences that were not accounted for by adjusting for imperfect proxy variables.

Second, existing immunoprophylaxis studies did not provide compelling evidence that RSV immunoprophylaxis protects against subsequent wheezing illness. As there were only eight studies (24 estimates) contributing to the model, our primary estimate of the effect of immunoprophylaxis on wheezing illness had a wide 95% CI (0·73–1·99), indicative of insufficient evidence for benefit or harm. Additional, preferably large, RCTs would improve precision.^97^ In sum, this study, in combination with previous analyses,^12^, ^13^ suggests that the evidence for a causal effect of RSV-LRTI on subsequent wheezing illness is not well supported by the existing data. Further, the current evidence does not support the assumption that effective RSV-LRTI prevention strategies would reduce subsequent wheezing illnesses.

Future observational studies evaluating the association between RSV-LRTI and wheezing illness are unlikely to be helpful in resolving the question of causality unless they accurately account for genetic influences. Additional twin registry studies would be valuable, particularly if the data could be combined to improve precision. Although we have focused on genetic risk as a potential confounder, future studies should also evaluate potential gene and RSV interactions.^11^, ^98^, ^99^ Studies measuring strong genetic markers (eg, 17q21 genotypes)^100^ could help to determine whether RSV has any meaningful causal effect, either independently or in combination with specific genotypes.

Regarding RSV immunoprophylaxis studies, RCTs are likely to provide less biased estimates than observational studies.^33^, ^35^ However, RCTs should evaluate the plausibility that randomised treatment assignment is a valid instrumental variable when drawing inferences about the causal effect of RSV-LRTI on wheezing illness.^24^ The case for randomised treatment assignment as an instrumental variable is predicated on making a compelling argument that RSV immunoprophylaxis only affects one's risk of wheezing illness by reducing the severity of RSV infection and not through any other mechanisms.^24^, ^101^ Powering a single RCT to detect effects on asthma in those aged 6 years or older will be challenging, owing to sample size requirements;^97^ however, combining data across multiple RCTs could improve precision. Standardisation of outcome measures should therefore be advocated when designing new RCTs.

Ultimately, evidence of causality in epidemiological research is always uncertain.^18^ However, studies can reduce uncertainty about whether RSV-LRTI causes wheezing illness by employing designs that minimise the influence of the most likely confounders^102^ and capitalising on causal modelling strategies.^17^, ^103^, ^104^ Uncertainty can also be reduced by use of objective markers of airway inflammation and disease (eg, lung function).^105^

The primary limitation of this Article is that it did not address all the evidence relevant to assessing whether RSV-LRTI causes wheezing illness. Several important studies did not meet our inclusion criteria. Notably, in a study of monozygotic and dizygotic twins, Thomsen and colleagues^12^ found that RSV-LRTI requiring treatment in hospital was more plausibly a marker of genetic risk rather than a cause of asthma, but the study was eliminated from our analysis because the RSV-LRTI exposure did not clearly precede the wheezing illness outcome.^12^ Wu and colleagues^8^ found that rates of asthma were correlated with birth near the peak of the winter RSV bronchiolitis season, consistent with a causal effect; however, this study was eliminated because it did not confirm RSV infection.^106^ Further, we did not review animal or human tissue studies experimentally evaluating mechanisms of RSV's effect on wheezing illness,^11^ nor did we include non-English language articles, which could have resulted in selection bias. Our findings should be interpreted in combination with these other important sources of evidence. The fact that all wheezing-related illnesses were combined into a single outcome in our analyses is another limitation, which could obscure effects on specific wheezing outcomes (eg, physician-diagnosed wheezing illness). Additionally, all RSV immunoprophylaxis studies have been done in high-risk populations, which could limit generalisability. Finally, nearly all the included data came from high-income countries; therefore, the findings might not be representative of low-income and middle-income countries.

This study had notable strengths. We used RVE meta-regression allowing for inclusion of multiple correlated estimates from the same study. Additionally, our search strategy was peer-reviewed by an expert information specialist. Finally, we provided all analytic code, allowing for replication and extension of our findings. Our assessment of findings from observational studies and immunoprophylaxis RCTs could neither discount a plausible non-causal model of the association between RSV-LRTI and subsequent wheezing illness, nor confirm that RSV immunoprophylaxis protects against wheezing illness. Consequently, this study does not provide compelling support for a causal effect of RSV-LRTI on subsequent wheezing illness. RSV-LRTI prevention will probably have a substantial public health effect by reducing complications associated with the acute infection;^107^ however, it remains uncertain whether there would be added value due to the prevention of recurrent wheezing illnesses.

## Data sharing

All analyses and R code are available at https://brunwasser.github.io/whorsv.github.io/.
