# Correction to *Lancet Respir Med* 2020; 8: 795–806

**DOI:** 10.1016/S2213-2600(20)30574-9

**Published:** 2021-01

*Brunwasser SM, Snyder BM, Driscoll AJ, et al. Assessing the strength of evidence for a causal effect of respiratory syncytial virus lower respiratory tract infections on subsequent wheezing illness: a systematic review and meta-analysis*. Lancet Respir Med *2020; **8:** 795–806*—This copyright has been changed to World Health Organization and should have been published under a CC BY 3.0 IGO license. This correction has been made to the online version as of Dec 11, 2020.

