# Supplementary material for: Assessing the strength of evidence for a causal effect of respiratory syncytial virus lower respiratory tract infections on subsequent wheezing illness: a systematic review and meta-analysis
Source: Lancet Respir Med. 2020 Aug;8(8):795–806. doi: 10.1016/S2213-2600(20)30109-0 (PMC7464591; doi:10.1016/S2213-2600(20)30109-0)
Supplement: Supplementary appendix [file mmc1.pdf]

THE LANCET
Respiratory Medicine

Supplementary appendix

This appendix formed part of the original submission and has been peer reviewed. We post it as supplied by the authors.

Supplement to: Brunwasser SM, Snyder BM, Driscoll AJ, et al. Assessing the strength of evidence for a causal effect of respiratory syncytial virus lower respiratory tract infections on subsequent wheezing illness: a systematic review and meta-analysis. *Lancet Respir Med* 2020; **8:** 795–806.

Online Supplementary Material

Assessing the strength of evidence for a causal effect of respiratory syncytial virus lower respiratory tract infection on subsequent wheezing illness: A systematic review and meta-analysis

Steven M. Brunwasser, Brittney M Snyder, Amanda J. Driscoll, Deshayne B. Fell, David A. Savitz, Daniel R. Feikin, Becky Skidmore, Niranjan Bhat, Louis J. Bont, William D. Dupont, Pingsheng Wu, Tebeb Gebretsadik, Patrick G. Holt, Heather J. Zar, *Justin R. Ortiz, *Tina V. Hartert

*Authors contributed equally to this submission

Corresponding author: Tina V. Hartert, tina.hartert@vumc.org, 1+(615) 936-1010

Table of Contents

Appendix E1. Final study protocol

1. INTRODUCTION

1.1. Background and motivation

1.2. Non-causal models of the RSV-LRTI wheezing illness and their testable implications

1.3. Role of RSV immunoprophylaxis trials in assessing the plausibility of a causal effect

1.4. Summary

2. METHOD

2.1. External review panel

2.2. Study selection criteria

2.3. Literature search

2.4. Identifying relevant articles

2.5. Data abstraction

2.6. Data structure

2.7. General analytic approach

3. DISCUSSION

3.1. Limitations

3.2. Strengths

4. MAJOR *POST-HOC* PROTOCOL REVISIONS

4.1 Combining all wheezing illness outcomes

4.2 Excluding comparisons with non-RSV-LRTI

4.3 Changing the primary analytic approach

4.4 Dropping secondary analyses

4.5 Flexible washout period

4.6 Aggregating over levels of effect modifiers

4.7 Mean effect size estimate for immunoprophylaxis studies without naïve estimates

4.8 Excluding conference abstracts

5. REFERENCES

Appendix E2. Final literature search criteria

Appendix E3. PRISMA checklist

Table E1. Newcastle-Ottawa Scale rating for risk of bias

Table E2. Cochrane Risk of Bias ratings for randomized controlled trials

Figure E1. Leave-one-out sensitivity analysis for RSV-LRTI exposure studies

APPENDIX E1. FINAL STUDY PROTOCOL

1. INTRODUCTION

1.1. Background and motivation

Respiratory syncytial virus lower respiratory tract infections (RSV-LRTI) and asthma are among the most common and important causes of respiratory morbidity in infancy and childhood throughout the world.[1,2] RSV is a common cause of acute LRTI, hospital admissions, and mortality in young children, resulting in a substantial global health burden.[1,2] Early life RSV infection has been associated with later asthma development,[3–5] the most common pediatric non-communicable disease,[6] affecting approximately 334 million people worldwide.[7] There is now compelling evidence that environmental influences, in addition to genetics, contribute substantially to asthma development.[8] Thus, primary asthma prevention is highly plausible if we can identify causal and modifiable early life disease processes.[3]

There is longstanding debate among airway scholars over the role of early life RSV-LRTI in the etiology of asthma.[5,9,10] Quantitative reviews have established a strong average association between RSV-LRTI exposure and subsequent wheezing and asthma,[11–13] but it remains unclear whether RSV-LRTIs are a causal contributor or merely a marker of airway disease susceptibility. This distinction is crucial for stakeholders appraising the financial viability and prudency of widespread implementation of RSV prevention programs. If RSV-LRTI were truly a causal agent and not merely a risk marker, then we would expect efficacious RSV immunoprophylaxis interventions (e.g., monoclonal antibodies and prenatal maternal vaccines) to reduce risk for pediatric asthma, greatly increasing their public health value.[14] Unfortunately, adequately powered randomized clinical trials (RCTs) evaluating the effect of RSV immunoprophylaxis on childhood asthma directly would require prohibitively large sample sizes (n > 6,000 per study arm).[15] We can, however, capitalize on the methodological heterogeneity of existing studies to evaluate the plausibility of causal and non-causal explanations for observed associations between RSV-LRTIs and asthma-related outcomes.

Our objective is to evaluate the extent to which there is compelling evidence for a causal effect of early life respiratory syncytial virus lower respiratory tract infection (RSV-LRTI) on pediatric asthma and wheezing illness. To that end, we conducted a systematic quantitative review of the literature, commissioned by the World Health Organization, estimating effects of early life RSV-LRTI and RSV immunoprophylaxis on subsequent wheezing and asthma. The purpose of the review is not to establish that an association between RSV-LRTI and asthma exists—this

has been established in prior meta-analyses[11–13]—but rather to evaluate the viability of causal and non-causal models and their testable implications.

1.2. Non-causal models of the RSV-LRTI wheezing illness and their testable implications

Our degree of confidence that RSV-LRTI contributes to asthma is dependent on the risk of bias in the existing literature, but also our ability to rule out alternative non-causal explanations. If studies that minimize plausible confounding influences yield effect sizes estimates that are considerably smaller than studies that do not, there would be evidence supportive of a non-causal association. In contrast, if effect size estimates are consistently positive and unrelated to whether potential confounding influences were minimized, this would provide evidence against non-causal models and lend credence to the hypothesis of a causal effect. We will consider the degree of evidence for the following non-causal alternative explanations:

Preexisting lung vulnerability hypothesis. It is plausible that observed associations between RSV-LRTI and pediatric wheezing illness are attributable to preexisting vulnerability to airway disease (both RSV-LRTI and wheezing illness) rather than a true causal effect. This diathesis may be due to (epi)genetic risk and/or early environmental exposures (including prenatal insults) that precede RSV-LRTI. This explanation has potentially testable implications that we will evaluate in this synthesis: Studies minimizing genetic confounding should have a smaller mean effect size associated with the effect of RSV-LRTI on wheezing illness relative to those that do not. Additionally, we expect that studies minimizing differences in measures of neonatal health (e.g., preterm birth) between RSV-LRTI exposure and comparators groups will also yield smaller effect sizes. Neonatal health measures are markers of perinatal insults that could contribute to risk for both RSV-LRTI and wheezing illness.

RSV-LRTI as a marker of other causal co-infections. Children susceptible to RSV-LRTI are likely susceptible to other airway infections. It is plausible that RSV-LRTI is simply a marker of a co-infection that is a true causal contributor. If this were true, we would expect studies minimizing the influence of co-infections to have mean effect size indicating no independent effect of RSV-LRTI. Additionally, there would be no increase in the effect size between non-RSV-LRTI and wheezing illness when RSV-LRTI is present as a co-infection compared to when RSV-LRTI is absent (i.e., no interaction between the RSV^- infection and the RSV^+ infection).

Fig 1 provides a directed acyclic graph (DAG) depicting potential non-causal mechanisms underlying the association between RSV-LRTI and wheezing illness. Unidirectional solid arrows represent presumed causal effects whereas the arrowless dotted line connecting RSV-LRTI and wheezing illness represents a spurious association. In

this model, a preexisting vulnerability to respiratory illness is the major confounder contributing causally to both the exposure and outcome of interest. Its confounding influence can be ameliorated by minimizing differences between the RSV-LRTI and comparator groups on factors contributing to respiratory illness diatheses (e.g., markers of genetic loading and perinatal insults) and/or variables presumed to be on the causal pathway from the preexisting respiratory diathesis to wheezing illness (e.g., non-RSV co-infections).

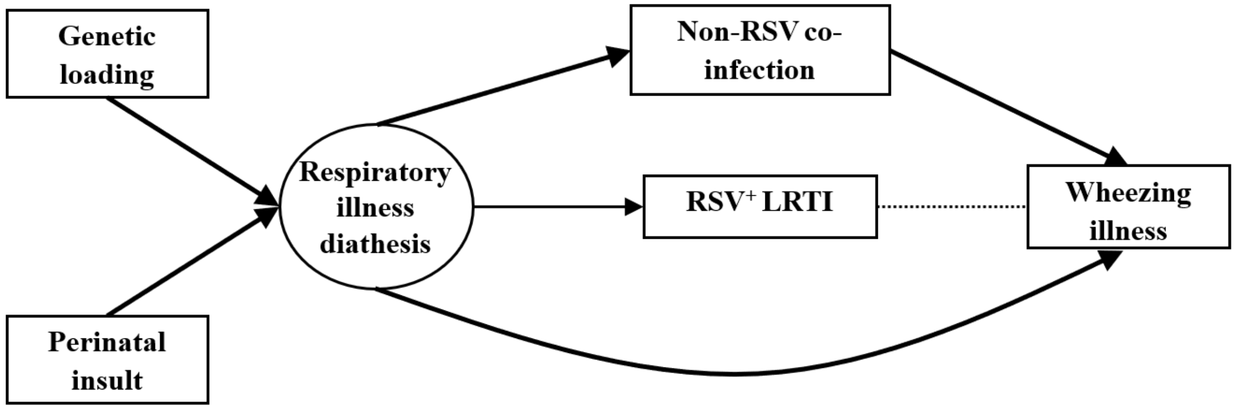

Fig 1. Directed acyclic graph depicting plausible non-causal pathways resulting in a spurious association of RSV-LRTI and subsequent wheezing illness. Causal effects are depicted with solid directional arrows and the spurious association is depicted with a non-directional dotted line. This model implies that effect sizes estimates will be smaller when they minimize the influence of (a) preexisting vulnerability to respiratory illness (and its causes) and (b) non-RSV co-infections.

1.3. Role of RSV immunoprophylaxis trials in assessing the plausibility of a causal effect

RSV immunoprophylaxis studies—particularly RCTs—provide information relevant to assessing the plausibility of RSV-LRTI as a causal contributor to subsequent wheezing illness. RCTs have shown that intramuscular injection of monoclonal antibodies (Palivizumab and Motavizumab) among infants during or just prior to seasons when RSV is circulating are efficacious in preventing RSV-LRTI or mitigating its effects.[16,17] If RSV-LRTI is a true cause of subsequent wheezing illness, it follows that—all things being equal—those receiving efficacious immunoprophylaxis should have reduced risk for wheezing illness. At present, RSV immunoprophylaxis is typically reserved for infants with serious health complications who would be most adversely affected by RSV infection.[18] Those receiving RSV immunoprophylaxis tend be substantially different in terms of their health status and likely their risk for wheezing illness (i.e., confounding by indication).[19] Consequently, non-randomized studies

evaluating the association between immunoprophylaxis studies under non-experimental conditions will likely only provide a minimally biased estimate effect of RSV-LRTI on wheezing illness if they minimize confounding by indication[20] and block all pathways from immunoprophylaxis receipt to wheezing illness that do not go through RSV-LRTI.[21]

Immunoprophylaxis RCTs allow for estimates of an unbiased causal effect under the condition that randomized condition assignment is an instrumental variable.[22] Unlike non-randomized studies, randomization ensures that any baseline differences between the treated and untreated groups are due to random chance rather than systematic features of the participants or experiment.[23] Perhaps, the most daunting assumption required for randomized immunoprophylaxis treatment condition to be an instrumental variable is the exclusion criterion,[22,24] which requires that randomized condition assignment influences wheezing illness *only indirectly* through its effect on the severity of RSV illness. That is, reducing risk/severity of RSV-LRTI is the only way in which immunoprophylaxis can affect wheezing illness. It is possible that assignment to the immunoprophylaxis condition could alter other processes on the causal pathway aside from the risk/severity of RSV-LRTI. For example, RSV immunoprophylaxis might result in increased risk of non-RSV infections that contribute to wheezing illness ("viral interference").[25,26] In this scenario, the association between randomized condition assignment and wheezing illness would yield an overly conservative estimate of the effect of RSV-LRTI and wheezing illness. If, on the other hand, immunoprophylaxis were to reduce the severity of other causal disease processes, we would obtain an inflated estimate.

Fig 2 depicts a DAG for a hypothetical immunoprophylaxis RCT where randomized condition assignment is an instrumental variable allowing for an unbiased estimate of the effect of RSV-LRTI on subsequent wheezing illness. In the DAG, x codes randomized condition assignment (e.g., 0=placebo, 1=immunoprophylaxis), m codes RSV-LRTI severity during infancy, and y codes subsequent wheezing illness. Path a depicts the effect of randomization to immunoprophylaxis on the risk/severity of RSV-LRTI. Path b depicts the effect of interest: the effect of RSV-LRTI on subsequent wheezing illness. If there is no pathway (c) connecting x and y without first going through RSV-LRTI (path a), then randomized condition assignment is a valid instrument.[22] This model also implies that randomized condition assignment and subsequent wheezing illness are independent when holding RSV-LRTI constant: $x \perp y|m$. This implication that could be disconfirmed in RCTs by testing whether the partial correlation between x and y adjusting for m equals 0 (or a small value that is practically equal to 0).[21]

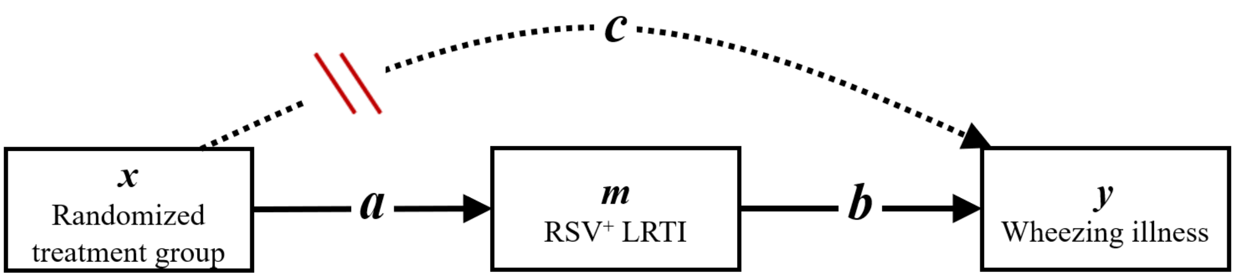

Fig 2. Directed acyclic graph depicting the exclusion criterion assumption necessary for randomized condition assignment to be an instrumental variable in an immunoprophylaxis RCT. The only pathway connecting randomized treatment group (*x*) and wheezing illness (*y*) must go through RSV-LRTI (*m*). There is no other pathway *c* that connects *x* and *y* without first going through *m*.

1.4. Summary

Our goal is to leverage the methodological heterogeneity of published data to evaluate the plausibility of causal and non-causal explanations for the established association between early life RSV-LRTI and subsequent wheezing illness. Although causality cannot be established with certainty, we aim to evaluate the tenability of alternative models using meta-analytic techniques and propose methods by which future studies could reduce the causal ambiguity.

2. METHOD

2.1. External review panel

At the outset of this project, we formed an external advisory group that has guided the development of this report. The group is comprised of scholars with relevant expertise and diverse skill sets. Advisory group members are tasked with reviewing all proposed procedures and outputs from this review and with helping to ensure that the investigators employ optimal procedures that minimize bias.

2.2. Study selection criteria

To be included in this review, studies have to measure **(a)** the direct association between RSV-LRTI and subsequent wheezing illness outcomes (RSV-LRTI *exposure studies*) and/or **(b)** the efficacy of RSV immunoprophylaxis on subsequent wheezing illness outcomes (*RSV prophylaxis studies*). We use the term *wheezing illness* to refer broadly to asthma or any other respiratory illnesses with wheezing episodes. Wheezing illness could be measured by physician diagnosis, parent/self-report, or research evaluation. Studies that do not measure wheezing

illness directly but measure only related constructs (e.g., like lung function or asthma related healthcare costs) will be excluded. Initially, we intended to develop separate meta-regression models for asthma and other wheezing illnesses (e.g., recurrent wheeze). However, studies did not use consistent definitions for these constructs. Some studies, for example, applied the label "asthma" to airway illnesses that were more consistent with what other studies termed "recurrent wheeze." Consequently, we combined all variations of wheezing illness into a single outcome variable in our analyses (see section 4.1). As described in section 2.7.4, we will conduct a subgroup analysis evaluating only estimates involving an asthma diagnosis occurring after age 5, when it is easier to distinguish between transient wheezing and persistent airway reactivity.[27]

The review will be limited to published (including E-pub ahead of print) peer-reviewed empirical studies. Ecological studies, case reports, clinical practice guidelines, commentaries, reviews, and animal studies will be excluded. There were insufficient translation resources available to include non-English articles in this synthesis. Table 1 summarizes all study inclusion and exclusion criteria.

Table 1. Summary of inclusion/exclusion criteria

	**RSV-LRTI Exposure Studies**	**RSV Immunoprophylaxis Studies**
**Article characteristics**	Empirical human subjects study published (including Epub ahead of print) in English in a peer-reviewed journal prior to final search date	
**Exposure**[a]	RSV-LRTI during a period beginning before age 2 and fully contained within ages 0-5 (operationalized as an exposure or mediator variable)	RSV immunoprophylaxis with established efficacy (either from the trial in question or past RCTs) in preventing/mitigating early life RSV-LRTI
**Comparator**	LRTI absent or undetected during the exposure period	RSV immunoprophylaxis not received during the exposure period
**Outcome**	Wheezing illness measured subsequent[b] to the index RSV-LRTI illness that defines membership in the exposure vs. comparator groups	Wheezing illness subsequent to study intervention protection period
**Sampling**	Exposure/comparator groups sampled from the same population	
**Exposure/outcome ascertainment**	Method of ascertaining exposure and outcomes were the same for exposure and comparator groups	

[a]Clinical trials may estimate the effect of RSV-LRTI on asthma/wheezing outcomes indirectly by reporting the effect of immunoprophylaxis on asthma/wheezing outcomes and/or report the direct association between RSV-LRTI and asthma/wheezing outcomes.
[b]Defined as occurring ≥ 30 days after the index RSV-LRTI.

2.2.1. *RSV-LRTI* exposure studies

2.2.1.1. *RSV-LRTI* exposure definition. We will include studies determining RSV exposure using laboratory techniques (e.g., real-time reverse transcriptase-polymerase chain reaction, serology, antigen testing, and viral culture), qualified medical evaluation, and/or medical records (e.g., diagnostic codes and RSV-related hospitalizations). Additionally, we will include studies estimating the effects of co-infection with RSV and other respiratory infections (e.g., influenza) as long as there is a comparator group that allowed for estimation of the incremental contribution of RSV-LRTI above the co-infection. Studies in which the exposure group is comprised of individuals with heterogeneous infections (e.g., RSV, rhinovirus, enterovirus, etc.) in which we cannot separate the unique contribution of RSV from other co-infections will be excluded.

The exposure for RSV-LRTI Exposure studies will be limited to RSV in the context of LRTI. Although RSV infection does not invariably result in lower respiratory symptoms, the public health impact of RSV is greatest in conjunction with LRTI.[28] Further, the most widely-studied immunoprophylaxis agent (e.g., palivizumab) is indicated for the explicit purpose of preventing RSV-induced LRTI in young children.[29] Although there has been recent work aimed at developing standard definitions for LRTI,[30] much of the relevant literature preceded these efforts. Consequently, we use a broad and flexible definition of LRTI to avoid eliminating relevant scholarship. Studies will be included if exposure group members have RSV (as defined above) in combination with LRTI conditions (e.g., bronchiolitis, pneumonia, acute bronchitis) or relevant clinical indications (e.g., wheeze, chest imaging, etc.). Finally, participants will be considered to have had an LRTI if they were hospitalized for an RSV-related illness.

Studies need to have an exposure ascertainment period beginning before two years of age and not extending beyond five years of age to be included; that is, the exposure period has to begin in the first two years of life ***and*** be fully contained within the first five years of life. For studies with an exposure window extending beyond age five, we will include estimates that are based on subgroup analyses evaluating the effect of RSV-LRTI within the allowable exposure period (e.g., evaluating the specific effect of RSV-LRTI between ages 0-5 on wheezing illnesses).

To ensure that the wheezing illness outcome was not merely a manifestation of the index LRTI (i.e., the outcome was separate from the exposure), we will require a "wash-out" period of 30 days between the index RSV-LRTI and measurement of the wheezing illness outcome. By 30 days post-illness onset, the vast majority of infected

infants and preschoolers have completed shedding RSV[31,32] and those with RSV bronchiolitis are no longer symptomatic.[33,34]

2.2.1.2. Comparator groups. Relevant comparator groups are those in which the criteria used to define the RSV-LRTI exposure group are absent, or present to a lesser degree (i.e., less severe illness), during the exposure window. Some studies determine exposure/comparator group status using viral surveillance in the first year of life: individuals with at least one RSV-LRTI are classified as members of the exposure group and those without an LRTI as the comparator group members (*viral surveillance* studies). Alternatively, some studies determine exposure/comparator group membership using a medical event (*medical event* studies). For example, individuals with a recorded hospitalization for RSV-LRTI during the ascertainment period are classified as members of the exposure group and those without as the comparator group. Some members of the comparator group in *medical event* studies likely had RSV infection in the exposure window, but did not have an infection severe enough to require medical attention. Additionally, studies comparing two groups with RSV-LRTI that differ only in the severity of their presentation are eligible for inclusion: e.g., studies comparing children with RSV-LRTI requiring emergency care or hospitalization (exposure group) to children with confirmed RSV-LRTI not requiring emergency care/hospitalization (comparator group).

2.2.2. RSV prophylaxis studies

2.2.2.1. RSV prophylaxis exposure and comparator group definitions. Studies will be included in this review if they report findings from a clinical trial (randomized or non-randomized) evaluating the efficacy of RSV immunoprophylaxis with participants who were either (a) administered RSV immunoprophylaxis directly between the ages 0-2, or (b) exposed *in utero* when the adult carrying the child during gestation received immunoprophylaxis. We will require that there was empirical evidence from at least one RCT that the prophylactic intervention in question is efficacious in targeting RSV in early childhood. The RSV prophylactic agent has to be compared to a comparator group that received no RSV immunoprophylaxis during the exposure period. We will also include estimates comparing infants who were more/less compliant with the immunoprophylaxis series: Those completing the full series of inoculations would be expected to have better protection from RSV-LRTI and thus, lower risk for wheezing illness relative to those completing only a partial series. At the time of this review, RSV immunoprophylaxis agents have only been approved for high-risk populations, including children with serious medical complications (e.g., preterm birth).[29] Thus, the findings of immunoprophylaxis studies may be relevant to

highly specific segments of the population and this will be reflected in our conclusions based on analysis of these trials.

2.3. Literature search

We conducted a comprehensive literature search of the MEDLINE and EMBASE databases for relevant scholarship. The search was an iterative process with collaboration between our subject-area experts and a master's level information specialist with expertise in systematic reviews. The original search parameters, delineated using the PRESS 2015 Guidelines,[35] were evaluated against a test-set of scholarly articles selected from recent reviews[3,36] and refined accordingly to ensure that the search was identifying articles with known relevance. An external master's level library and information specialist conducted an independent review of the search strategy and recommended minor revisions. A summary of the full search sequence and the final search parameters are provided in Appendix E2.

2.4. Identifying relevant articles

Four study investigators, all with doctoral degrees in healthcare sciences or epidemiology, reviewed the records identified in our final literature search in two stages. Stage 1 was a rapid review of all abstracts by two independent reviewers. Any article that was deemed potentially relevant (e.g., by mentioning RSV, LRTI, and/or any airway outcomes) was retained for full-text review (Stage 2). Reviewers were instructed to be liberal in Stage 1 and only exclude articles for which they had a high degree of confidence that inclusion criteria were not met. If there was a discrepancy among reviewers in Stage 1 (i.e., one reviewer eliminated the article and the other did not), the article was retained for full-text review.

In the Stage 2 full-text review, two independent reviewers determined whether all articles surviving the rapid review met the inclusion criteria and would be included in the synthesis. Reviewers terminated full-text reviews once a single exclusion criterion was identified rather than checking each inclusion/exclusion criterion individually. Discrepancies among reviewers in Stage 2 were resolved by consensus.

2.5. Data abstraction

Data relevant to the meta-analysis will be abstracted from the included articles into a database by an investigator with a doctoral degree in either in epidemiology or a healthcare field. An independent doctoral level investigator will review the data from each article for quality assurance purposes, resolving discrepancies by consensus.

2.5.1. Effect size estimates

The odds ratios (OR) is the most commonly reported effect size in this review; therefore, our analyses quantify differences between RSV-LRTI exposure and comparator groups using the natural log of the odds ratio: log_e(OR). Standard errors for estimates of interest are rarely reported and thus we will calculate it in one of two ways. When a 95% confidence interval is provided, we will calculate the *SE* as:

$$SE_{log_e OR} = [log_e(CI_U) - log_e(OR)]/1.96, \quad (1)$$

where CI_U is the upper bound of the 95% confidence interval of the OR estimate. When only descriptive data are available (i.e., the number of exposed and comparator group members with and without wheezing illness), we will calculate standard errors for the log_e(OR) as follows:[37]

$$SE_{log_e OR} = \sqrt{(1/a) + (1/b) + (1/c) + (1/d)}, \quad (2)$$

where *a* and *b* represent the number of RSV-LRTI exposed participants with and without the wheezing illness outcome, respectively; and *c* and *d* are the number of comparator group participants with and without the wheezing illness outcomes, respectively.

Some of the effect sizes included in this review will be based on analyses adjusting for potential confounders, whereas others will be naïve estimates. We will use unadjusted estimates of the effect of immunoprophylaxis on wheezing illness for RCTs because appropriate randomization procedures ensure unbiasedness:[23] any differences between the treated and untreated are due to chance rather than features of the experiment. For *RSV-LRTI exposure* studies and non-randomized immunoprophylaxis studies, we will include estimates adjusting for potential confounders (aOR) in our analyses rather than unadjusted estimates whenever possible, as the former are generally expected to be less biased. Some observational studies adjust for covariates representing processes that clearly followed the RSV-LRTI exposure temporally and are plausibly on the causal pathway. This could lead to overly conservative estimates as a potential causal pathway through the mediator is blocked.[21] We will conduct sensitivity analyses removing both naïve estimates (i.e., no effort to minimize confounding) and those that adjusted for likely mediators (see section 2.7.5).

Some studies report adjusted effect size estimates other than the aOR, including adjusted risk ratios (aRR), hazard ratios (aHR), and incident rate ratios (aIRR). Rather than excluding these estimates or calculating unadjusted

ORs based on descriptive statistics, we will treat them as conservative approximations of the OR. This strategy biases toward the null because these alternative estimates are smaller than the OR.[38]

2.5.2. Study characteristics and effect modifiers

In addition to effect size data, we will code key study features to summarize the included scholarship and to serve as effect modifiers in our meta-regression models. The income level of the countries in which studies were conducted will be determined using the World Bank classification: https://datahelpdesk.worldbank.org/knowledgebase/articles/906519-world-bank-country-and-lending-groups. We will code whether the study used a targeted enrollment strategy requiring all participants to have a known risk factor (other than RSV-LRTI) for wheezing illness (e.g., family history of atopy). The time intervals covered by outcome assessments differ considerably across studies. Some effect sizes code prevalence of wheezing illness outcomes (e.g., in the past year), whereas others coded incidence over a specific time period. Consequently, we cannot code mean/median age-at-outcome as a precise continuous time variable. Rather, we will create a categorical variable coding whether the outcome assessed wheezing primarily in the preschool years (mean/median age < 5), primary school years (mean/median age 5-12); adolescence (mean/median age 13-18), or adulthood mean/median > 18). Finally, because the timing of the RSV-LRTI exposure may moderate its impact on wheezing illness, we will code whether the exposure ascertainment period was fully contained in the first year of life or whether it extended beyond the first year.

Of particular importance, we will code the extent to which studies attempted to minimize the impact of potential key confounders, including genetic predisposition for wheezing illness, neonatal health proxies (e.g., Apgar scores), and co-infections. Investigators will code whether the influence of these potential confounders was likely minimized *not at all*, *somewhat*, or *mostly/completely*. Minimization of potential bias could be achieved by study design (e.g., randomization or matching), statistical analysis (e.g., covariate adjustment or propensity scores), or chance (i.e., confounders well-balanced at baseline between the exposure and comparator groups).

2.5.3. Risk of bias ratings

In order to have a general sense of the degree to which the estimates included in this review accurately reflect their population parameters, we will rate each effect size for risk of bias using established instruments: the Newcastle-Ottawa Scale (NOS)[39] for observational studies (including non-randomized immunoprophylaxis studies)

and the Cochrane Risk of Bias Tool (CRBT)[40] for immunoprophylaxis RCTs. Both tools are freely available online.[1] We will use these ratings to help identify the most common bias threats in studies estimating the effect of RSV-LRTI on wheezing illness and corresponding priorities for improving future study design and methodology.

For the NOS, coders rate the risk of bias on the following seven study features: representativeness of the exposed cohort; selection of the non-exposed cohort; ascertainment of the exposure; demonstration that the presence of the outcome did not precede study onset; comparability of the exposed & unexposed groups; ascertainment of the outcome; adequacy of the follow-up period (i.e., sufficient time for outcomes to occur); and retention of participants at follow-ups. The CRBT tool evaluates the following areas: selection bias, performance bias, detection bias, attrition bias, reporting bias, and other biases not covered by the other areas. For many studies, it is necessary to code risk of bias separately for different outcomes and/or comparisons. For example, the risk of bias due to response variable missingness could be considerable for one outcome (e.g., laboratory-based tests) and minimal for another (e.g., medical record asthma diagnosis).

There are several important things to note about the NOS risk of bias ratings.

- *Item 1. Representativeness of the exposed cohort*. The top rating states that the exposed group was "truly representative" of the community of interest. It is often unclear what the parameters of the "community" are and to whom the investigators were hoping to draw inferences. We reserved the top rating "truly representative" for studies including everyone in a well-defined population, or large samples from multiple well-defined populations.
- *Item 3. Selection of Controls*: RSV-LRTI is almost invariably determined using molecular methods or hospital records based on molecular methods. We will rate molecular determination as "Secure record."
- *Item 4. Definition of Controls*: This item asks whether the outcome of interest was absent at the study outset. Generally, among RSV-LRTI exposure studies, only longitudinal birth cohort studies monitoring infants closely from birth could determine with high confidence whether participants had some form of wheezing illness prior to the study outset. In studies measuring wheezing illness outcome as the occurrence of wheezing-related medical events, we cannot be certain that infants did not have wheezing illness symptoms that did not elicit medical attention prior to study outset. For the purposes of this study, we will

[1] The NOS scale is available at: http://www.ohri.ca/programs/clinical_epidemiology/oxford.asp. The CRBT is available at: https://sites.google.com/site/riskofbiastool/welcome/rob-2-0-tool?authuser=0.

code “yes” for this item if the participants had no evidence of the outcome of interest (e.g., medically-attended wheeze) prior to the study onset, with the understanding that we cannot know with certainty whether illness manifestations were present before the study period.

2.6. Data structure

Most studies in this review provided multiple, non-independent effect size estimates. Many studies reported multiple exposure-comparator group comparisons, either with multiple RSV-LRTI exposure groups compared to the same comparator group, or multiple comparator groups compared to the same RSV-LRTI group. Others reported effect sizes for multiple correlated outcomes (e.g., physician-diagnosed asthma and parent-reported wheeze), often across repeated measurement occasions. To make optimal use of the available data and avoid effect size selection bias, we included all relevant estimates that provided at least some unique information in our primary analyses. If a study provided effect sizes estimating the association between RSV-LRTI and wheezing illness outcomes in the full population *and* effect sizes for specific subgroups within that population, we included only the former in our primary analyses because the latter provided no unique information.

2.7. General analytic approach

2.7.1. Meta-analytic assumptions and modeling technique

Studies contributing to this review draw participants from heterogeneous populations and used varied research methodology. Consequently, a fixed-effects meta-analysis, which assumes that all contributing studies estimate a common true population effect size (β_0), is untenable. Rather, we will use a random-effects modeling framework, assuming that each study estimates a true effect sizes for a unique population (β_0^j), with each β_0^j conceptualized as being drawn from a theoretical distribution of true population effect sizes. Each β_0^j deviates by a certain amount (u^j) from an overall mean population effect size (γ_{00}).[41] Random-effects models include two variance parameters that sum to the total variance: the variance of the within-study random sampling errors, $e^{ij} \sim N(0, \sigma^2)$, and between-study deviations around γ_{00}, $u^j \sim N(0, \tau^2)$.

To accommodate our data’s complex correlation structure arising from within-study dependencies, we will use robust variance estimation (RVE) meta-regressions[42] using the robumeta package version 2.0[43] in R version 3.5.0.[44] The RVE approach adjusts coefficient standard errors in the presence of dependent estimates to provide confidence intervals with good coverage probabilities.[42,45] Traditional meta-analysis assumes that all effect sizes estimates are independent, i.e., all off-diagonal elements of the effect size correlation matrix (**Σ**) are equal to 0.

When studies provide multiple dependent estimates, each study requires its own effect size correlation matrix ($\boldsymbol{\Sigma^j}$) in which the covariance elements are not constrained to 0. Multilevel meta-analysis, an alternative approach for dependent correlation structures, requires that all elements of the $\boldsymbol{\Sigma^j}$ are treated as known and within-study dependencies are modeled explicitly.[45,46] However, studies rarely report sufficient information (e.g., correlations among dependent variables) to provide sound estimates of within-study covariances.[45,46] A major advantage of the RVE approach is that it does not require knowledge of the within-study correlation structure to yield accurate standard errors. It uses the cross-products of residuals for a given study *j* ($\boldsymbol{e^j e^{j\prime}}$) to approximate the unknown covariance elements of $\boldsymbol{\Sigma^j}$.[42] Simulations studies have shown that the RVE approach yields both unbiased model parameter estimates and confidence interval coverage probabilities close to their nominal levels,[42] particularly when used in conjunction with small-sample-size adjustments.[46,47]

The RVE approach focuses on making accurate inferences for fixed-effects covariates and not on quantifying the degree of heterogeneity in the populations of effect sizes.[45] Thus, our analyses will focus on fixed-effects estimates and not on variance parameters. However, an estimate of the between-study variability ($\hat{\tau}^2$) is needed in order to calculate approximate inverse variance weights, which are more efficient than other weighting strategies.[42] As recommended by the RVE developers,[42,43] we will use a simple working model of the within-study effect size correlation structure in which a common correlation (ρ) is applied to all off-diagonal elements of $\boldsymbol{\Sigma^j}$.[47] We will set ρ = 0.80 in our primary models, reflecting the assumption that effect sizes from the same study are highly correlated, though the value of ρ generally has little impact on parameter and imprecision estimates.[42,47] In sensitivity analyses, we will evaluate whether selecting alternative values of ρ alters conclusions in any meaningful way (see section 2.7.5). Once we have an estimate of $\hat{\tau}^2$, we will then calculate approximate inverse variance weights for each effect size estimate *i* within study *j* using the formula provided by Hedges et al[42]:

$$w^{ij} = \frac{1}{k^j(v_{.}^j + \hat{\tau}^2)}, \quad (5)$$

where $v_{.}^j$ is the mean of the within-study variance for all of the k^j effect sizes in study *j*, and $\hat{\tau}^2$ is the estimate of between-study variability. As recommended by Tipton (2015) to avoid inflated Type I error rates, we will apply small-sample-size adjustments to the sampling variances of model regression coefficients, regardless of the number of observations contributing to the models, and use the Satterthwaite approximation for calculating degrees of freedom (*df*).[47]

2.7.2. Model-building approach

A primary challenge in this review is statistically combining estimates from heterogeneous studies to obtain meaningful aggregate estimates.[41] This is particularly challenging for *RSV+ exposure* studies and non-randomized *RSV immunoprophylaxis* studies because we use—whenever possible—model-based effect size estimates that limit potential confounders rather than naïve estimates. Studies rarely use identical models to obtain these estimates and, consequently, they do not have precisely the same meaning. However, we conceptualize all estimates as imperfect approximations of the effect of RSV-LRTI on wheezing illness, with some estimates being more biased than others. We attempt to account for heterogeneity in effect size estimates in two ways: (a) dividing estimates into more homogeneous clusters for analyses,[48] and (b) including model covariates to account for major potential sources of heterogeneity.

In terms of analyzing more homogenous subgroups, we will run separate meta-regression models for *RSV+ Exposure* studies and *RSV immunoprophylaxis* studies. Both study types are relevant when evaluating the plausibility of a causal effect of RSV-LRTI on subsequent wheezing illness, but they differ considerably in terms of their primary research questions, methods, and threats to causal inference. For example, confounding by indication[20] is uniquely relevant to the non-randomized prophylaxis trials. Therefore, a meta-regression model for prophylaxis studies likely requires a different functional form than observational studies of RSV-LRTI exposure. Finally, among the *RSV-LRTI exposure* studies, we will run several in pre-specified subgroup analyses (described in section 2.7.5), evaluating the effects of RSV-LRTI among specific sub-populations.

We also attempt to account for heterogeneity in effect size estimates by including covariates (i.e., effect size moderators) in our meta-regression models. Covariates for meta-regression analyses were selected *a priori* based on substantive considerations. First and foremost, we selected covariates that address our primary study hypotheses. For example, in the *RSV-LRTI exposure* regression model, we include covariates coding whether or not any effort was made to limit the potential confounding influence of genetic predisposition, neonatal health markers, and co-infections. Additionally, we include covariates representing important study design features that could plausibly account for effect size heterogeneity, improving precision and interpretability, but whose inclusion is not driven by a primary hypothesis. Some model covariates (x^{ij}) measure characteristics that vary both within and between studies (e.g., age at outcome ascertainment), while others (w^{j}) measured study-level characteristics that are constant within-studies (e.g., risk- vs. non-risk-based enrollment strategy).

In accordance with recommendations by Tipton,[47] regression coefficient estimates with Satterthwaite $df < 4$ will be considered unreliable, as confidence intervals for these estimates tend to be overly liberal. In these situations, we will seek to identify potential causes that were not captured in preliminary data screening (including high leverage data points, unbalanced covariates, and sparse data cells[47]) and, when indicated, rerun the model after applying corrective procedures (e.g., aggregating cells of a categorical covariate). Additionally, we will run sensitivity analyses for all models, as described in section 2.7.5.

2.7.3. Primary meta-regression models

2.7.3.1. Model 1: *RSV-LRTI exposure* studies. The purpose of this model is to test the hypotheses that, among *RSV-LRTI exposure* studies, effect sizes will be smaller when minimizing the influence of genetic predisposition, neonatal health proxies, and co-infections. A secondary hypothesis is that effect sizes estimates will be largest among outcomes measured during the preschool years, in close proximity to the RSV-LRTI exposure, relative to outcomes measured later in life. Thus, we will include the categorical covariate representing age-at-outcome. We will also adjust for covariates coding study design features that could plausibly account for effect size heterogeneity: risk- vs. non-risk-based enrollment strategy and exposure ascertainment windows (limited to the first 12 months of life or not). We expect that effect size estimate would be larger among studies using a risk-based enrollment strategy and whose exposure ascertainment was limited to the first year of life, though these are not primary hypotheses. The model includes two interactions, allowing both the effect of age at outcome ascertainment and the exposure ascertainment window to differ across *medical event* and *viral surveillance* studies.

The model has the following form:

$$y^{ij} = \gamma_{00} + \gamma_{10}x_1^{ij} + \gamma_{20}x_2^{ij} + \gamma_{30}x_3^{ij} + \gamma_{40}x_4^{ij} + \gamma_{50}x_5^{ij} + \gamma_{01}w_1^{j} + \gamma_{02}w_2^{j} + \gamma_{60}\left(x_1^{ij} \times x_2^{ij}\right) + \gamma_{11}\left(x_1^{ij} \times w_2^{j}\right) + e^{ij} + u^{j} \qquad (6)$$

where y^{ij} = $\log_e$(OR) for effect size estimate *i* within study *j*; γ_{00} = conditional mean of the distribution of population effect sizes; $\gamma_{10} \cdots \gamma_{60}$ = regression weights for covariates (x^{ij}) whose values vary within studies; γ_{01}, γ_{02} = regression weights for covariates (w^{j}) whose values are constant within studies; γ_{11}= regression weight for a cross-level interaction; e^{ij} = within-study sampling error; u^{j} = between-study deviations from the population average effect size. The model covariates are defined as follows:

- x_1^{ij} = *study design*: 0=exposure group membership determined by RSV-LRTI medical event; 1= exposure group membership determined by viral surveillance
- x_2^{ij} = *age at outcome ascertainment*: 0=preschool years (mean/median age < 5); 1=primary school years (mean/median age ≥ 5); 2=adolescence/adulthood (mean/median age ≥ 13)
- x_3^{ij} = *genetic confounding*: 0=no attempt to reduce potential genetic confounding; 1=confounding reduced at least somewhat
- x_4^{ij} = *co-infection confounding*: 0=no attempt to reduce potential confounding due to co-infections at time of RSV-LRTI ascertainment; 1= confounding reduced at least somewhat
- x_5^{ij} = *neonatal health confounding*: 0=no attempt to reduce potential confounding due to infant neonatal health; 1= confounding reduced at least somewhat
- w_1^j = *participant risk*: 0=study included children regardless of risk status; 1=study included only children at risk for wheezing illness beyond having an RSV-LRTI;
- w_2^j = *exposure ascertainment period*: 0=study exposure ascertainment extends beyond 12 months of age; 1= exposure ascertainment entirely within the first 12 months of life.

2.7.3.2. Model 2: RSV immunoprophylaxis studies. In this model, we test the hypothesis that those receiving RSV immunoprophylaxis—and who presumably have lower risk/severity of RSV-LRTI—will have lower odds of subsequent wheezing illness. As there are relatively few *RSV immunoprophylaxis* studies, the model will be simpler than the model for *RSV-LRTI exposure* studies by necessity. The model takes the following form:

$$y^{ij} = \gamma_{00} + \gamma_{01} w_1^j + e^{ij} + u^j, \quad (7)$$

where w_1^j codes whether the study was an RCT or non-randomized prophylaxis study and the other parameters are as defined in equation (6). Of particular interest in *RSV immunoprophylaxis* studies is the extent to which estimates were based on models limiting confounding by indication.

2.7.4. Subgroup analyses

In addition to our primary analyses, we will run several subgroup analyses evaluating effect size estimates in among subpopulations that share key characteristics. First, we will conduct a meta-regression including only effect size estimates for asthma, determined as part of the study protocol or through medical records, measured at age ≥ 6 years. This analysis will help us determine whether effect of RSV-LRTI on wheezing illness are driven

primarily by transient wheezing outcomes. Second, some airway scholars have suggested that the effect of early life viral infections on wheezing illness may be limited to—or more pronounced among—individuals with a genetic diathesis.[49] Therefore, we will run analyses among the subgroup of estimates that were derived from samples with and without a genetic vulnerability for wheezing illness. This is typically determined by asking whether the child had a family history of asthma or atopy. Finally, there is some evidence that the effect of infant viral infection is moderated by whether the child has atopic sensitization,[50] though the evidence for this is stronger for human rhinovirus than RSV.[51] We will conduct a meta-regression among the subpopulation with established atopic sensitization by age 2 evaluating the effect of RSV-LRTI on wheezing illness after ascertainment of atopic sensitization.

2.7.5. Sensitivity analyses

We will conduct several forms of sensitivity analyses. First, we will evaluate whether results are sensitive to the value of our within-study common correlation value ($\rho = 0.80$) used to approximate inverse variance weights, as described in section 2.7.1. We will rerun each model five times, substituting ρ with values ranging from 0.00 to 1.00 at intervals of 0.20, evaluating whether parameter and standard error estimates change meaningfully. Second, in order to evaluate the influence of individual studies on the overall results, we will rerun each model while iteratively removing one study (and all of its effect sizes) at a time. Finally, we will rerun models removing all naïve effect size estimates (where no attempt was made to minimize confounding) and all adjusted estimates that controlled for potential mediators at the effect of RSV-LRTI on wheezing illness. The former estimates are expected to be upwardly biased whereas the latter are expected to be downwardly biased.

3.0 DISCUSSION

3.1. Limitations

The results of this synthesis should be interpreted with full awareness of several important limitations. First, an inherent limitation of meta-analysis using summary statistics reported in manuscripts is that we work with incomplete data. Participant-level data contains important information (e.g., covariance among outcomes) that is typically unavailable to the meta-analyst. Second, owing to insufficient translation resources, we limited our review to the English language literature, which may limit the generalizability of our findings. Third, we rely on peer-reviewed publications for our data analysis. It may be that studies finding a statistically significant association between RSV-LRTI and wheezing illness outcomes are more likely to be published, resulting in an upward bias in

our effect estimates, though this is not invariably the case.[52] However, our sensitivity analyses will help gauge how likely it is that unpublished scholarship might have altered our conclusions. We considered including unpublished abstracts in the review, but these records rarely had sufficient information to determine with confidence whether they met the inclusion criteria or to be included in meta-analytic models. Additionally, many described in-progress research which had not undergone thorough peer-review, potentially introducing other sources of bias.[53] Finally, our review only includes human-subjects studies; evidence from animal model research can help establish a biological plausibility and inform potential causal pathways that can be disconfirmed in human subjects research.[54]

3.2. Strengths

This review also has several notable strengths. First, it will be, to our knowledge, the most comprehensive analytic evaluation of the association between RSV-LRTI and wheezing illness, including both observational studies and immunoprophylaxis RCTs. Second, we approach the review with the aim of testing specific non-causal hypotheses which could undermine the argument that the association is causal. Furthermore, we delineate the assumptions required to make causal inferences for various study designs and make recommendations for how future research can explicitly test and potentially disconfirm causal implications from their hypothesized models. Third, our methods were reviewed in advance by a panel of scholars with unique expertise in areas relevant to this review. Fourth, we used modeling strategies that allow for the retention of multiple correlated effect sizes from single studies while providing confidence intervals that closely approximate their nominal levels. This likely reduces the risk of biased effect size estimate selection and loss of information. Finally, we provide all data and statistical code as supplements for use by other scholars.

4.0. MAJOR *POST-HOC* PROTOCOL REVISIONS

A challenge in developing a meta-analytic protocol is that we cannot know whether all of our planned methods and analyses are feasible until we have abstracted the data. There may be insufficient data, for instance, to support a planned analysis. Here we summarize major protocol revisions that took place after we began data abstraction and our rationale for making them.

4.1 Combining all wheezing illness outcomes

Originally, we planned to conduct analyses evaluating the effects of RSV-LRTI on asthma separately from other wheezing illnesses. However, owing to the fact that definitions of asthma were inconsistent across studies we

combined all wheezing illnesses into a single outcome. However, we conducted a secondary analysis limited to outcomes described as "asthma" by study authors and measured at age ≥ 6 years old when it becomes easier to distinguish between transient and chronic wheezing illness.

4.2 Excluding comparisons with non-RSV-LRTI

Our original protocol did not exclude estimates comparing RSV-LRTI to non-RSV-LRTI. However, these comparisons do not have clear implications for whether RSV-LRTI is a cause of subsequent wheezing illness and were consequently excluded. The primary question of interest in terms of estimating causality is whether children would be at greater risk for subsequent wheezing illness if they had an early life RSV-LRTI compared to the counterfactual in which they did not have an early life RSV-LRTI. The comparison of RSV-LRTI to non-RSV-LRTI addresses a different question: whether there is a relative difference in the effects of RSV-LRTI compared to an LRTI with a different etiology. If RSV-LRTI were causal, this would **not** imply that the mean effect size comparing RSV-LRTI to non-RSV-LRTI should be positive (RSV-LRTI has stronger association with wheezing illness), null, or negative (non-RSV-LRTI has a stronger association). All of these outcomes are plausible regardless of whether RSV is causal or not. In sum, there are no clear causal implications from this comparison. A recent meta-analysis found that the association between RSV-LRTI and subsequent wheezing illness is less strong than the association between non-RSV-LRTI and wheezing illness,[13] but this does not help us determine whether the association between RSV-LRTI and subsequent wheezing illness is causal.

4.3 Changing the primary analytic approach

We originally planned to conduct random-effects meta-analyses using the Hartung-Knapp approach [55,56] However, it became clear as we reviewed articles that many studies measured multiple relevant wheezing illness outcomes, had multiple relevant exposure and/or comparator groups, and/or measured outcomes repeatedly over time. In order to make full use of the available data rather than selecting specific outcomes or averaging them, we needed an approach that could accommodate complex correlation structures among effect size estimates. Therefore, we switched to the Robust Variance Estimation (RVE) method.[42] which can accommodate studies that provided multiple correlated outcomes even when the within-study correlations are unknown.[45–47]

4.4 Dropping secondary analyses

We originally proposed a secondary analysis evaluating the potential modifying influence of early atopic sensitization on the effect of RSV-LRTI on subsequent wheezing illness.[50] We planned to stratify our primary

analyses looking specifically within subgroups of children with and without confirmed early life (by age 2) atopic sensitization. However, too few studies provided relevant data to support these analyses. Additionally, we planned to evaluate the subgroup of studies conducted within low- and middle-income countries; however, all but two studies were conducted in high-income countries, precluding meaningful subgroup analyses. **4.5. Flexible washout period**

Our initial protocol required that there be a 30-day washout period between the RSV-LRTI exposure and measurement of the wheezing illness outcome. For many effect estimates, it was not possible to determine with certainty that all measured wheezing illnesses captured in the outcome ascertainment occurred after the washout period. For example, some studies assessed at follow-up assessment whether the child had ever had any wheezing episodes in the time since the index RSV-LRTI. We chose to include these studies because we expect that only a small minority of ascertained episodes occurred within the 30-day washout period and we did not want to lose relevant information. However, we excluded studies in which most or all of the outcome ascertainment period occurred during the washout period: e.g., studies only measuring wheezing illnesses in the month following the index episode. Additionally, we conducted a sensitivity analysis including only studies in which it was clear that all illness episodes occurred outside of the washout period.

4.6. Aggregating over levels of effect modifiers

As described in section 2.5.2, we coded age-at-outcome as a categorical effect modifier with four levels: Preschool age (median/mean age 0-4 years old); School age (5-12); Adolescence (13-18); and Adulthood (19+). As there were relatively few estimates from the adolescent and adulthood years, we combined these categories in our analyses to avoid sparse cells.

Similarly, owing to sparse cells, effect modifiers coding the extent to which effect size estimates were based on analyses limiting confounders were treated as binary in study analyses. These effect modifiers originally had three levels: *not at all*, *somewhat*, and *mostly/completely*. We aggregated over the *somewhat* and *mostly/completely* levels to create binary variables.

4.7. Mean effect size estimate for immunoprophylaxis studies without naïve estimates

For two of the studies[57,58] contributing to the RSV immunoprophylaxis analyses, there was no explicit effort to adjust for potential group differences in preexisting health factors. Consequently, we reran our primary RSV immunoprophylaxis model with estimates from these two studies excluded. Thus, in addition to our primary

analysis including all RSV immunoprophylaxis studies, we ran a secondary analysis including only studies making explicit attempts to reduce confounding by indication.

4.8. Excluding conference abstracts

We initially intended to include conference abstracts from 2016-2018 in the review. However, when reviewing conference abstracts identified in our literature search, it became clear that many did not provide sufficient information to determine with adequate conference whether the study satisfied the inclusion criteria. Additionally, many described in-progress research which had not undergone thorough peer-review, potentially introducing other sources of bias.[53] Consequently, conference abstracts were excluded from the review.

5. REFERENCES

1 Scheltema N, Gentile A, Lucion F, *et al.* Global respiratory syncytial virus-associated mortality in young children (RSV GOLD): a retrospective case series. *Lancet Glob Health* 2017; **5**: e984–91.

2 Shi T, McAllister DA, O'Brien KL, *et al.* Global, regional, and national disease burden estimates of acute lower respiratory infections due to respiratory syncytial virus in young children in 2015: a systematic review and modelling study. *Lancet* 2017; **390**: 946–58.

3 Feldman AS, He Y, Moore ML, Hershenson MB, Hartert TV. Toward primary prevention of asthma. Reviewing the Evidence for early-life respiratory viral infections as modifiable risk factors to prevent childhood asthma. *Am J Respir Crit Care Med* 2015; **191**: 34–44.

4 Jackson D, Hartert T, Martinez F, Weiss S, Fahy J. Asthma: NHLBI workshop on the primary prevention of chronic lung diseases. *Ann Am Thorac Soc* 2014; **11 Suppl 3**: S139-145.

5 Wu P, Hartert TV. Evidence for a causal relationship between respiratory syncytial virus infection and asthma. *Expert Review of Anti-infective Therapy* 2011; **9**. http://informahealthcare.com/doi/abs/10.1586/eri.11.92 (accessed Dec 15, 2015).

6 Asher I, Pearce N. Global burden of asthma among children. *The international journal of tuberculosis and lung disease* 2014; **18**: 1269–1278.

7 Global Asthma Network. The global asthma report 2014. Auckland, New Zealand, 2014.

8 Burbank A, Sood A, Kesic M, Peden D, Hernandez M. Environmental determinants of allergy and asthma in early life. *J Allergy Clin Immunol* 2017; **140**: 1–12.

9 Kuehni CE, Spycher BD, Silverman M. Causal links between RSV infection and asthma. *Am J Respir Crit Care Med* 2009; **179**: 1079–80.

10 Thomsen SF, Stensballe LG. Respiratory syncytial virus and asthma in twin children: lessons from the population-based Danish registries. *Current Respiratory Medicine Reviews* 2011; **7**: 167–171.

11 Kabego L, de Beer C. Association between respiratory syncytial virus infection in infancy and subsequent asthma: A meta-analysis of observational studies. *JSM Allergy and Asthma* 2017; **2**: 1009.

12 Régnier S, Huels J. Association between respiratory syncytial virus hospitalizations in infants and respiratory sequelae: Systematic review and meta-analysis. *Pediatr Infect Dis J* 2013; **32**: 820–6.

13 Shi T, Ooi Y, Zaw EM, *et al.* Association between respiratory syncytial virus-associated acute lower respiratory infection in early life and recurrent wheeze and asthma in later childhood. *J Infect Dis* 2019; **[Epub ahead of print]**. DOI:10.1093/infdis/jiz311.

14 Abreo A, Gebretsadik T, Stone Jr C, Hartert T. The impact of modifiable risk factor reduction on childhood asthma development. *Clinical and Translational Medicine* Accepted for publication.

15 Riddell CA, Bhat N, Bont LJ, *et al.* Informing randomized clinical trials of respiratory syncytial virus vaccination during pregnancy to prevent recurrent childhood wheezing: A sample size analysis. *Vaccine* 2018; **36**. DOI:10.1016/j.vaccine.2018.10.041.

16 Feltes TF, Sondheimer HM, Tulloh RMR, *et al.* A randomized controlled trial of motavizumab versus palivizumab for the prophylaxis of serious respiratory syncytial virus disease in children with hemodynamically significant congenital heart disease. *Pediatric Research* 2011; **70**: 186–91.

17 Gutfraind A, Galvani AP, Meyers LA. Efficacy and optimization of palivizumab injection regimens against respiratory syncytial virus infection. *JAMA Pediatr* 2015; **169**: 341–348.

18 Wegzyn C, Toh LK, Notario G, *et al.* Safety and Effectiveness of Palivizumab in Children at High Risk of Serious Disease Due to Respiratory Syncytial Virus Infection: A Systematic Review. *Infect Dis Ther* 2014; **3**: 133–58.

19 Carroll KN, Gebretsadik T, Escobar GJ, *et al.* Respiratory syncytial virus immunoprophylaxis in high-risk infants and development of childhood asthma. *J Allergy Clin Immunol* 2017; **139**: 66-71.e3.

20 Kyriacou DN, Lewis RJ. Confounding by indication in clinical research. *JAMA* 2016; **316**: 1818–9.

21 Pearl J, Glymour M, Jewell N. Causal inference in statistics: a primer. Chichester, West Sussex, The United Kingdom: John Wiley & Sons, 2016.

22 Angrist JD, Imbens GW, Rubin DB. Identification of Causal Effects Using Instrumental Variables. *Journal of the American Statistical Association* 1996; **91**: 444–55.

23 Deaton A, Cartwright N. Understanding and misunderstanding randomized controlled trials. *Soc Sci Med* 2017; **210**: 2–21.

24 Baiocchi M, Cheng J, Small DS. Instrumental variable methods for causal inference. *Statist Med* 2014; **33**: 2297–2340.

25 Blanken M, Rovers M, Molenaar J, *et al.* Respiratory syncytial virus and recurrent wheeze in healthy preterm infants. *New England Journal of Medicine* 2013; **368**: 1791–9.

26 Achten NB, Wu P, Bont L, *et al.* Interference between respiratory syncytial virus and human rhinovirus infection in infancy. *J Infect Dis* 2017; **215**: 1102–6.
27 Martinez FD, Wright AL, Taussig LM, Holberg CJ, Halonen M, Morgan WJ. Asthma and wheezing in the first six years of life. *New England Journal of Medicine* 1995; **332**: 133–8.
28 Nair H, Nokes DJ, Gessner BD, *et al.* Global burden of acute lower respiratory infections due to respiratory syncytial virus in young children: a systematic review and meta-analysis. *The Lancet* 2010; **375**: 1545–55.
29 Committee on Infectious Diseases and Committee on Fetus and Newborn. Revised indications for the use of palivizumab and respiratory syncytial virus immune globulin intravenous for the prevention of respiratory syncytial virus infections. *Pediatrics* 2003; **112**: 1442–6.
30 Greene G, Hood K, Little P, *et al.* Towards clinical definitions of lower respiratory tract infection (LRTI) for research and primary care practice in Europe: an international consensus study. *Primary Care Respiratory Journal* 2011; **20**: 299–306.
31 Munywoki PK, Koech DC, Agoti CN, *et al.* Influence of age, severity of infection, and co-infection on the duration of respiratory syncytial virus (RSV) shedding. *Epidemiology & Infection* 2015; **143**: 804–12.
32 Hall CB, Douglas RG, Geiman JM. Respiratory syncytial virus infections in infants: Quantitation and duration of shedding. *The Journal of Pediatrics* 1976; **89**: 11–5.
33 Petruzella FD, Gorelick MH. Duration of illness in infants with bronchiolitis evaluated in the emergency department. *Pediatrics* 2010; **126**: 285–90.
34 Swingler GH, Hussey GD, Zwarenstein M. Duration of Illness in ambulatory children diagnosed with bronchiolitis. *Arch Pediatr Adolesc Med* 2000; **154**: 997–1000.
35 McGowan J, Sampson M, Salzwedel DM, Cogo E, Foerster V, Lefebvre C. PRESS peer review of electronic search strategies: 2015 guideline statement. *Journal of Clinical Epidemiology* 2016; **75**: 40–6.
36 Jin J, Zhou Y-Z, Gan Y-C, Song J, Li W-M. Early-life respiratory infections are pivotal in the progression of asthma-a systematic review and meta-analysis. *Int J Clin Exp Med* 2017; **10**: 4256–66.
37 Bland JM, Altman DG. The odds ratio. *BMJ* 2000; **320**: 1468.
38 Cook TD. Advanced statistics: up with odds ratios! A case for odds ratios when outcomes are common. *Acad Emerg Med* 2002; **9**: 1430–4.
39 Wells GA, Shea B, O'Connell D, *et al.* The Newcastle-Ottawa Scale (NOS) for assessing the quality of nonrandomised studies in meta-analyses. The Ottawa Hospital Research Institute. http://www.ohri.ca/programs/clinical_epidemiology/oxford.asp (accessed July 30, 2019).
40 Higgins JPT, Altman DG, Gøtzsche PC, *et al.* The Cochrane Collaboration's tool for assessing risk of bias in randomised trials. *BMJ* 2011; **343**: d5928.
41 Borenstein M, Hedges L, Rothstein H. Meta-analysis: fixed effect vs. random effects. 2007. http://www.meta-analysis.com/downloads/Meta-analysis%20fixed%20effect%20vs%20random%20effects.pdf (accessed Oct 26, 2014).
42 Hedges LV, Tipton E, Johnson MC. Robust variance estimation in meta-regression with dependent effect size estimates. *Research Synthesis Methods* 2010; **1**: 39–65.
43 Fisher Z, Tipton E. robumeta: An R-package for robust variance estimation in meta-analysis. 2015; published online March 7. http://arxiv.org/abs/1503.02220 (accessed May 13, 2019).
44 R Core Team. R: A language and environment for statistical computing. Vienna, Austria: R Foundation for Statistical Computing, 2018 https://www.R-project.org/.
45 Tanner-Smith EE, Tipton E, Polanin JR. Handling complex meta-analytic data structures using robust variance estimates: A tutorial in R. *J Dev Life Course Criminology* 2016; **2**: 85–112.
46 Moeyaert M, Ugille M, Beretvas SN, Ferron J, Bunuan R, Van den Noortgate W. Methods for dealing with multiple outcomes in meta-analysis: a comparison between averaging effect sizes, robust variance estimation and multilevel meta-analysis. *International Journal of Social Research Methodology* 2017; **20**: 559–72.
47 Tipton E. Small sample adjustments for robust variance estimation with meta-regression. *Psychol Methods* 2015; **20**: 375–93.
48 Savitz DA, Wellenius GA. Interpreting epidemiologic evidence: connecting research to applications, 2nd edn. New York, NY, US: Oxford University Press, 2016.
49 Gern JE. The ABCs of rhinoviruses, wheezing, and asthma. *Journal of Virology* 2010; **84**: 7418–26.
50 Kusel MMH, de Klerk NH, Kebadze T, *et al.* Early-life respiratory viral infections, atopic sensitization, and risk of subsequent development of persistent asthma. *Journal of Allergy and Clinical Immunology* 2007; **119**: 1105–10.
51 Jackson DJ, Evans MD, Gangnon RE, *et al.* Evidence for a causal relationship between allergic sensitization and rhinovirus wheezing in early life. *Am J Respir Crit Care Med* 2012; **185**: 281–5.

52 Schmucker CM, Blümle A, Schell LK, *et al.* Systematic review finds that study data not published in full text articles have unclear impact on meta-analyses results in medical research. *PLoS One* 2017; **12**. DOI:10.1371/journal.pone.0176210.
53 Higgins JPT, Green S, editors. Cochrane handbook for systematic reviews of interventions, Version 5.1.0 [updated March 2011]. The Cochrane Collaboration, 2011 www.handbook.cochrane.org.
54 Han J, Takeda K, Gelfand EW. The role of RSV infection in asthma initiation and progression: Findings in a mouse model. *Pulmonary Medicine* 2011; **Article ID 748038**. DOI:10.1155/2011/748038.
55 Röver C, Knapp G, Friede T. Hartung-Knapp-Sidik-Jonkman approach and its modification for random-effects meta-analysis with few studies. *BMC Medical Research Methodology* 2015; **15**: 99.
56 Hartung J, Knapp G. On tests of the overall treatment effect in meta-analysis with normally distributed responses. *Statistics in Medicine* 2001; **20**: 1771–82.
57 dos Santos Simões MCR, Inoue Y, Matsunaga NY, *et al.* Recurrent wheezing in preterm infants: Prevalence and risk factors. *Jornal de Pediatria* 2018; **[Epub ahead of print]**. DOI:10.1016/j.jped.2018.06.007.
58 Prais D, Kaplan E, Klinger G, *et al.* Short-and long-term pulmonary outcome of palivizumab in children born extremely prematurely. *Chest* 2016; **149**: 801–8.

APPENDIX E2: LITERATURE SEARCH CRITERIA

2018 Aug 14 search

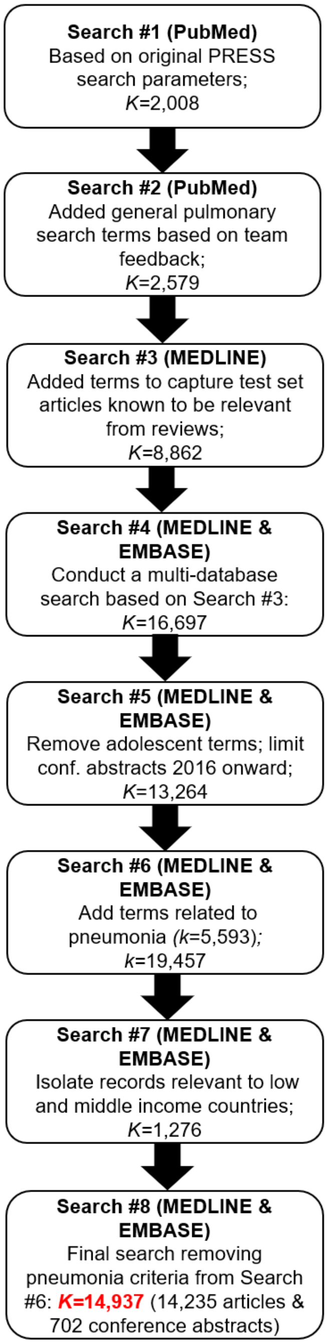

Ovid Multifile

Database: Embase Classic+Embase <1947 to 2018 August 13>, Ovid MEDLINE(R) ALL <1946 to August 13, 2018>
Search Strategy:
----
1 Respiratory Syncytial Viruses/ (5714)
2 Respiratory Syncytial Virus, Human/ (4477)
3 Respiratory Syncytial Virus Infections/ (7371)
4 (respiratorysync#tial virus* or respiratory sync#tial virus* or sync#tial respiratory virus*).tw,kf. (26560)
5 (respiratorysync#tial pneumovirus* or respiratory sync#tial pneumovirus* or sync#tial respiratory pneumovirus*).tw,kf. (0)
6 (HRSV or RSV).tw,kf. (24996)
7 RS virus*.tw,kf. (1151)
8 sync#tial virus*.tw,kf. (26730)
9 Respiratory Syncytial Virus Vaccines/ (1728)
10 anti-RSV.tw,kf. (681)
11 motavizumab.tw,kf. (108)
12 ("medi 524" or medi524 or numax).tw,kf. (104)
13 Palivizumab/ (3129)
14 (palivizumab or abbosynagis or "MEDI 493" or MEDI493 or synagis or synagys).tw,kf. (2599)
15 or/1-14 [RSV EXPOSURE] (39482)
16 Respiratory Tract Infections/ (54746)
17 Respiratory Tract Diseases/ (44909)
18 (airway? adj2 infect*).tw,kf. (5871)
19 (respiratory tract? adj2 infect*).tw,kf. (49274)
20 (LRTI or LRTIs).tw,kf. (2792)
21 (LRI or LRIs).tw,kf. (1092)
22 (respiratory adj (illness* or infect*)).tw,kf. (60222)
23 Bronchiolitis/ (15148)
24 bronchiolitis.tw,kf. (24848)
25 ((airflow* or air flow* or lung function*) adj2 deficit?).tw,kf. (263)
26 or/16-25 [RESPIRATORY INFECTIONS - GENERAL] (204433)
27 15 or 26 [RSV, RESPIRATORY INFECTIONS] (229386)
28 exp Infant/ (2114208)
29 exp Child/ (4453138)
30 (infant or infants or infanc* or baby or babies or boy or boys or child* or girl or girls or preschool* or pre-school* or toddler*).tw,kf. (3868206)
31 Pediatrics/ (122883)
32 (pediatric* or paediatric*).tw,kf. (761389)
33 (newborn* or neonat* or premie?).tw,kf. (829930)
34 (very low birth weight* or VLBW or VLBWs or "small for gestational age" or "small for gestational ages" or SGA or SGAs).tw,kf. (43942)
35 or/28-34 [INFANT/CHILD POPULATION] (6441847)
36 27 and 35 (88317)
37 exp Asthma/ (361999)
38 asthma*.tw,kf. (354212)
39 (bronchospasm* or broncho-spasm* or bronchial spasm*).tw,kf. (13419)
40 Respiratory Sounds/ (11407)
41 wheez*.tw,kf. (32226)
42 Bronchial Hyperreactivity/ (17886)

43 Respiratory Hypersensitivity/ (14009)
44 ((respirat* or airway or airways or lung or lungs or bronchi* or broncho* or bronchu*) adj3 (sensitivit* or hypersensitivit* or hyper-sensitivit* or hyperreactiv* or hyper-reactiv*)).tw,kf. (18975)
45 exp Hypersensitivity/ (922059)
46 (allergy or allergies or allergic*).tw,kf. (384435)
47 Lung Injury/ (38071)
48 ((lung or lungs) adj3 injur*).tw,kf. (65480)
49 ((airway or airways or lung or lungs or respirat*) adj5 (patholog* or histopatholog* or histo-patholog*)).tw,kf. (40161)
50 exp Respiratory System/pa [Pathology] (96416)
51 or/37-50 [ASTHMA/WHEEZE/LRTIs] (1282966)
52 36 and 51 [RSV - INFANT/CHILD - ASTHMA/WHEEZE/LRTIs] (20019)
53 exp Animals/ not (exp Animals/ and Humans/) (16453732)
54 52 not 53 [ANIMAL-ONLY REMOVED] (15306)
55 (comment or editorial or news or newspaper article).pt. (1788984)
56 (letter not (letter and randomized controlled trial)).pt. (1968033)
57 54 not (55 or 56) [OPINION PIECES REMOVED] (14918)
58 57 use medall [MEDLINE RECORDS] (8162)
59 pneumovirus/ (203)
60 exp human respiratory syncytial virus/ (4565)
61 respiratory syncytial virus infection/ (9583)
62 (respiratorysync#tial virus* or respiratory sync#tial virus* or sync#tial respiratory virus*).tw,kw. (26841)
63 (respiratorysync#tial pneumovirus* or respiratory sync#tial pneumovirus* or sync#tial respiratory pneumovirus*).tw,kw. (0)
64 (HRSV or RSV).tw,kw. (25147)
65 RS virus*.tw,kw. (1162)
66 sync#tial virus*.tw,kw. (26875)
67 respiratory syncytial virus vaccine/ (1728)
68 anti-RSV.tw,kw. (682)
69 motavizumab/ (221)
70 motavizumab.tw,kw. (110)
71 ("medi 524" or medi524 or numax).tw,kw. (104)
72 palivizumab/ (3129)
73 (palivizumab or abbosynagis or "MEDI 493" or MEDI493 or synagis or synagys).tw,kw. (2639)
74 or/59-73 [RSV EXPOSURE] (39459)
75 respiratory tract infection/ (92367)
76 lower respiratory tract infection/ (9629)
77 respiratory tract disease/ (76306)
78 (airway? adj2 infect*).tw,kw. (5927)
79 (respiratory tract? adj2 infect*).tw,kw. (48661)
80 (LRTI or LRTIs).tw,kw. (2804)
81 (LRI or LRIs).tw,kw. (1098)
82 (respiratory adj (illness* or infect*)).tw,kw. (60504)
83 exp bronchiolitis/ (27087)
84 bronchiolitis.tw,kw. (25381)
85 ((airflow* or air flow* or lung function*) adj2 deficit?).tw,kw. (263)
86 or/75-85 [RESPIRATORY INFECTIONS - GENERAL] (258656)
87 74 or 86 [RSV, RESPIRATORY INFECTIONS] (282469)
88 exp child/ (4453138)
89 (infant or infants or infanc* or baby or babies or boy or boys or child* or girl or girls or preschool* or pre-school* or toddler*).tw,kw. (3862285)
90 exp Pediatrics/ (156577)
91 (pediatric* or paediatric*).tw,kw. (780424)
92 (newborn* or neonat* or premie?).tw,kw. (825466)
93 (very low birth weight* or VLBW or VLBWs or "small for gestational age" or "small for gestational ages" or SGA or SGAs).tw,kw. (44580)

94 or/88-93 [INFANT/CHILD POPULATION] (6283150)
95 87 and 94 (100602)
96 exp asthma/ (361999)
97 asthma*.tw,kw. (358521)
98 (bronchospasm* or broncho-spasm* or bronchial spasm*).tw,kw. (13582)
99 abnormal respiratory sound/ (6827)
100 wheezing/ (30841)
101 wheez*.tw,kw. (32457)
102 bronchus hyperreactivity/ (11950)
103 respiratory tract allergy/ (9771)
104 ((respirat* or airway or airways or lung or lungs or bronchi* or broncho* or bronchu*) adj3 (sensitivit* or hypersensitivit* or hyper-sensitivit* or hyperreactiv* or hyper-reactiv*)).tw,kw. (19372)
105 hypersensitivity/ (88422)
106 (allergy or allergies or allergic*).tw,kw. (389619)
107 lung injury/ (38071)
108 ((lung or lungs) adj3 injur*).tw,kw. (66733)
109 ((airway or airways or lung or lungs or respirat*) adj5 (patholog* or histopatholog* or histo-patholog*)).tw,kw. (40046)
110 or/96-109 [ASTHMA/WHEEZE/LRTIs] (911092)
111 95 and 110 [RSV - INFANT/CHILD - ASTHMA/WHEEZE/LRTIs] (21511)
112 exp animal/ or exp animal experimentation/ or exp animal model/ or exp animal experiment/ or nonhuman/ or exp vertebrate/ (47551256)
113 exp human/ or exp human experimentation/ or exp human experiment/ (36484992)
114 112 not 113 (11067938)
115 111 not 114 [ANIMAL-ONLY REMOVED] (20900)
116 editorial.pt. (1004022)
117 letter.pt. not (letter.pt. and randomized controlled trial/) (1963354)
118 115 not (116 or 117) [OPINION PIECES REMOVED] (20379)
119 conference abstract.pt. (3109222)
120 118 not 119 [CONFERENCE ABSTRACTS REMOVED] (17757)
121 120 use emczd [EMBASE RECORDS] (10530)
122 58 or 121 [BOTH DATABASES] (18692)
123 limit 122 to yr="2012-current" (5224)
124 remove duplicates from 123 (3796)
125 limit 122 to yr="2004-2011" (5648)
126 remove duplicates from 125 (3923)
127 limit 122 to yr="1990-2003" (4986)
128 remove duplicates from 127 (3470)
129 122 not (123 or 125 or 127) (2834)
130 remove duplicates from 129 (2075)
131 124 or 126 or 128 or 130 [TOTAL UNIQUE RECORDS] (13264)
132 131 use medall [MEDLINE UNIQUE RECORDS] (8097)
133 131 use emczd [EMBASE UNIQUE RECORDS] (5167)

****

Conference Abstracts – 2016-2018 (*Embase only*)

Database: Embase Classic+Embase <1947 to 2018 August 13>
Search Strategy:
----
1 pneumovirus/ (54)
2 exp human respiratory syncytial virus/ (2454)
3 respiratory syncytial virus infection/ (3447)
4 (respiratorysync#tial virus* or respiratory sync#tial virus* or sync#tial respiratory virus*).tw,kw. (14611)
5 (respiratorysync#tial pneumovirus* or respiratory sync#tial pneumovirus* or sync#tial respiratory pneumovirus*).tw,kw. (0)

6 (HRSV or RSV).tw,kw. (14190)
7 RS virus*.tw,kw. (722)
8 sync#tial virus*.tw,kw. (14694)
9 respiratory syncytial virus vaccine/ (1188)
10 anti-RSV.tw,kw. (368)
11 motavizumab/ (221)
12 motavizumab.tw,kw. (61)
13 ("medi 524" or medi524 or numax).tw,kw. (85)
14 palivizumab/ (2478)
15 (palivizumab or abbosynagis or "MEDI 493" or MEDI493 or synagis or synagys).tw,kw. (1782)
16 or/1-15 [RSV EXPOSURE] (22629)
17 respiratory tract infection/ (56347)
18 lower respiratory tract infection/ (9629)
19 respiratory tract disease/ (55152)
20 (airway? adj2 infect*).tw,kw. (3629)
21 (respiratory tract? adj2 infect*).tw,kw. (29165)
22 (LRTI or LRTIs).tw,kw. (1656)
23 (LRI or LRIs).tw,kw. (622)
24 (respiratory adj (illness* or infect*)).tw,kw. (35978)
25 exp bronchiolitis/ (19206)
26 bronchiolitis.tw,kw. (15304)
27 ((airflow* or air flow* or lung function*) adj2 deficit?).tw,kw. (155)
28 or/17-27 [RESPIRATORY INFECTIONS - GENERAL] (164697)
29 16 or 28 [RSV, RESPIRATORY INFECTIONS] (178113)
30 exp child/ (2669695)
31 (infant or infants or infanc* or baby or babies or boy or boys or child* or girl or girls or preschool* or pre-school* or toddler*).tw,kw. (2184318)
32 exp Pediatrics/ (102884)
33 (pediatric* or paediatric*).tw,kw. (467555)
34 (newborn* or neonat* or premie?).tw,kw. (469365)
35 (very low birth weight* or VLBW or VLBWs or "small for gestational age" or "small for gestational ages" or SGA or SGAs).tw,kw. (25541)
36 or/30-35 [INFANT/CHILD POPULATION] (3482301)
37 29 and 36 (60610)
38 exp asthma/ (241860)
39 asthma*.tw,kw. (212165)
40 (bronchospasm* or broncho-spasm* or bronchial spasm*).tw,kw. (8436)
41 abnormal respiratory sound/ (6827)
42 wheezing/ (22589)
43 wheez*.tw,kw. (19908)
44 bronchus hyperreactivity/ (11950)
45 respiratory tract allergy/ (9771)
46 ((respirat* or airway or airways or lung or lungs or bronchi* or broncho* or bronchu*) adj3 (sensitivit* or hypersensitivit* or hyper-sensitivit* or hyperreactiv* or hyper-reactiv*)).tw,kw. (11328)
47 hypersensitivity/ (41957)
48 (allergy or allergies or allergic*).tw,kw. (238272)
49 lung injury/ (32015)
50 ((lung or lungs) adj3 injur*).tw,kw. (39030)
51 ((airway or airways or lung or lungs or respirat*) adj5 (patholog* or histopatholog* or histo-patholog*)).tw,kw. (23555)
52 or/38-51 [ASTHMA/WHEEZE/LRTIs] (560874)
53 37 and 52 [RSV - INFANT/CHILD - ASTHMA/WHEEZE/LRTIs] (13965)
54 exp animal/ or exp animal experimentation/ or exp animal model/ or exp animal experiment/ or nonhuman/ or exp vertebrate/ (25828855)
55 exp human/ or exp human experimentation/ or exp human experiment/ (19250536)
56 54 not 55 (6579372)

57 53 not 56 [ANIMAL-ONLY REMOVED] (13514)
58 editorial.pt. (538292)
59 letter.pt. not (letter.pt. and randomized controlled trial/) (971114)
60 57 not (58 or 59) [OPINION PIECES REMOVED] (13152)
61 conference abstract.pt. (3109222)
62 60 and 61 [CONFERENCE ABSTRACTS] (2622)
63 limit 62 to yr="2016-current" (600)

2018 Aug 28 search focused on identifying studies from low- and middle- income countries and measuring pneumonia

MEDLINE

Database: Ovid MEDLINE(R) and Epub Ahead of Print, In-Process & Other Non-Indexed Citations and Daily <1946 to August 27, 2018>
Search Strategy:
----
1 Respiratory Syncytial Viruses/ (5669)
2 Respiratory Syncytial Virus, Human/ (2118)
3 Respiratory Syncytial Virus Infections/ (6143)
4 (respiratorysync#tial virus* or respiratory sync#tial virus* or sync#tial respiratory virus*).tw,kf. (12269)
5 (respiratorysync#tial pneumovirus* or respiratory sync#tial pneumovirus* or sync#tial respiratory pneumovirus*).tw,kf. (0)
6 (HRSV or RSV).tw,kf. (11014)
7 RS virus*.tw,kf. (440)
8 sync#tial virus*.tw,kf. (12356)
9 Respiratory Syncytial Virus Vaccines/ (541)
10 anti-RSV.tw,kf. (315)
11 motavizumab.tw,kf. (49)
12 ("medi 524" or medi524 or numax).tw,kf. (19)
13 Palivizumab/ (652)
14 (palivizumab or abbosynagis or "MEDI 493" or MEDI493 or synagis or synagys).tw,kf. (860)
15 or/1-14 [RSV EXPOSURE] (17489)
16 Respiratory Tract Infections/ (36059)
17 Respiratory Tract Diseases/ (21173)
18 (airway? adj2 infect*).tw,kf. (2315)
19 (respiratory tract? adj2 infect*).tw,kf. (21318)
20 (LRTI or LRTIs).tw,kf. (1153)
21 (LRI or LRIs).tw,kf. (482)
22 (respiratory adj (illness* or infect*)).tw,kf. (25314)
23 Bronchiolitis/ (3006)
24 bronchiolitis.tw,kf. (10191)
25 ((airflow* or air flow* or lung function*) adj2 deficit?).tw,kf. (108)
26 or/16-25 [RESPIRATORY INFECTIONS - GENERAL] (93674)
27 15 or 26 [RSV, RESPIRATORY INFECTIONS] (104425)
28 exp Infant/ (1073142)
29 exp Child/ (1785279)
30 (infant or infants or infanc* or baby or babies or boy or boys or child* or girl or girls or preschool* or pre-school* or toddler*).tw,kf. (1707884)
31 Pediatrics/ (49811)
32 (pediatric* or paediatric*).tw,kf. (314596)
33 (newborn* or neonat* or premie?).tw,kf. (368538)
34 (very low birth weight* or VLBW or VLBWs or "small for gestational age" or "small for gestational ages" or SGA or SGAs).tw,kf. (19134)
35 or/28-34 [INFANT/CHILD POPULATION] (2979066)
36 27 and 35 (41327)
37 exp Asthma/ (120295)
38 asthma*.tw,kf. (146695)
39 (bronchospasm* or broncho-spasm* or bronchial spasm*).tw,kf. (5158)
40 Respiratory Sounds/ (8272)
41 wheez*.tw,kf. (12596)
42 Bronchial Hyperreactivity/ (7210)
43 Respiratory Hypersensitivity/ (9287)

44 ((respirat* or airway or airways or lung or lungs or bronchi* or broncho* or bronchu*) adj3 (sensitivit* or hypersensitivit* or hyper-sensitivit* or hyperreactiv* or hyper-reactiv*)).tw,kf. (8198)
45 exp Hypersensitivity/ (323993)
46 (allergy or allergies or allergic*).tw,kf. (155772)
47 Lung Injury/ (6067)
48 ((lung or lungs) adj3 injur*).tw,kf. (28248)
49 ((airway or airways or lung or lungs or respirat*) adj5 (patholog* or histopatholog* or histo-patholog*)).tw,kf. (17254)
50 exp Respiratory System/pa [Pathology] (96512)
51 or/37-50 [ASTHMA/WHEEZE/LRTIs] (540544)
52 36 and 51 [RSV - INFANT/CHILD - ASTHMA/WHEEZE/LRTIs] (8660)
53 exp Animals/ not (exp Animals/ and Humans/) (4491073)
54 52 not 53 [ANIMAL-ONLY REMOVED] (8414)
55 (comment or editorial or news or newspaper article).pt. (1252689)
56 (letter not (letter and randomized controlled trial)).pt. (993558)
57 54 not (55 or 56) [OPINION PIECES REMOVED] (8169)
58 exp Pneumonia/ (85837)
59 pneumoni*.tw,kf. (163129)
60 58 or 59 [PNEUMONIA] (193513)
61 35 and 60 (44180)
62 51 and 61 (4887)
63 62 not 53 (4635)
64 63 not (55 or 56) (4552)
65 64 not 57 [UNIQUE PNEUMONIA REFS] (3131)
66 Developing Countries.sh,kf. (81580)
67 (Africa? or Asia? or Caribbean or West Indies or South America? or Latin America? or Central America?).hw,kf,tw,cp. (483167)
68 (Afghanistan or Albania or Algeria or Angola or Antigua or Barbuda or Argentina or Armenia or Armenian or Aruba or Azerbaijan or Bahrain or Bangladesh or Barbados or Benin or Byelarus or Byelorussian or Belarus or Belorussian or Belorussia or Belize or Bhutan or Bolivia or Bosnia or Herzegovina or Hercegovina or Botswana or Brasil or Brazil or "Brazzavilee" or Bulgaria or Burkina Faso or Burkina Fasso or Upper Volta or Burundi or Urundi or Cambodia or Khmer Republic or Kampuchea or Cameroon or Cameroons or Cameron or Camerons or "Canary Islands" or Cape Verde or Central African Republic or Chad or Chile or China or Colombia or Comoros or Comoro Islands or Comores or Mayotte or Congo or Zaire or Costa Rica or Cote d'Ivoire or Ivory Coast or Croatia or Cuba or Cyprus or Czechoslovakia or Czech Republic or Slovakia or Slovak Republic or Djibouti or French Somaliland or Dominica or Dominican Republic or East Timor or East Timur or Timor Leste or Ecuador or Egypt or United Arab Republic or El Salvador or Eritrea or Estonia or Ethiopia or Fiji or Gabon or Gabonese Republic or Gambia or Gaza or Georgia Republic or Georgian Republic or Ghana or Gold Coast or Greece or Grenada or Guatemala or Guinea or Guam or Guiana or Guyana or Haiti or Honduras or Hungary or India or Maldives or Indonesia or Iran or Iraq or Isle of Man or Jamaica or Jamahiriya? or Jamahiryia? or Jordan or Kazakhstan or Kazakh or Kenya or Kiribati or Korea or Kosovo or Kyrgyzstan or Kirghizia or Kyrgyz Republic or Kirghiz or Kirgizstan or Lao PDR or Laos or Latvia or Lebanon or Lesotho or Basutoland or Liberia or Libia or Libya or Lithuania or Macedonia or Madagascar or Maghreb or Maghrib or Malagasy Republic or Malaysia or Malaya or Malay or Mayote or Mocambique or Principe or Reunion or Sabah or Sarawak or Malawi or Nyasaland or Mali or Malta or Marshall Islands or Mauritania or Mauritius or Agalega Islands or Mexico or Micronesia or Middle East or Moldova or Moldovia or Moldovian or Mongolia or Montenegro or Morocco or Ifni or Mozambique or Myanmar or Myanma or Burma or Namibia or Nepal or Netherlands Antilles or New Caledonia or Nicaragua or Niger or Nigeria or Northern Mariana Islands or Oman or Muscat or Pakistan or Palau or Palestine or Panama or Paraguay or Peru or Philippines or Philipines or Phillipines or Phillippines or Poland or Portugal or Puerto Rico or Romania or Rumania or Roumania or Russia or Russian or Rwanda or Ruanda or Saint Kitts or St Kitts or Nevis or Saint Lucia or St Lucia or Saint Vincent or St Vincent or Grenadines or Samoa or Samoan Islands or Navigator Island or Navigator Islands or "St Helena" or "Saint Helena" or Sao Tome or Saudi Arabia or Senegal or Serbia or Montenegro or Seychelles or Sierra Leone or Slovenia or Sri Lanka or Ceylon or Solomon Islands or Somalia or South Africa or Sudan or Suriname or Surinam or Swaziland or Syria or Tajikistan or Tadzhikistan or Tadjikistan or Tadzhik or Tanzania or Thailand or Togo or Togolese Republic or Tonga or Trinidad or Tobago or Tunisia or Turkey or Turkmenistan or Turkmen or Uganda or Ukraine or Uruguay or USSR or Soviet Union or Union of Soviet Socialist Republics or Uzbekistan or

Uzbek or Vanuatu or New Hebrides or Venezuela or Vietnam or Viet Nam or West Bank or "Western Sahara" or "Western Saharan" or Yemen or Yugoslavia or Zambia or Zimbabwe or Rhodesia).hw,kf,ti,ab,cp. (3379588)
69 ((developing or less* developed or under developed or underdeveloped or middle income or low* income or underserved or under served or deprived or poor*) adj (countr* or nation? or population? or world)).tw,kf. (114113)
70 ((developing or less* developed or under developed or underdeveloped or middle income or low* income) adj (economy or economies)).tw,kf. (443)
71 (low* adj (gdp or gnp or gross domestic or gross national)).tw,kf. (216)
72 (low adj3 middle adj3 countr*).tw,kf. (11445)
73 (lmic or lmics or third world or lami countr*).tw,kf. (5868)
74 transitional countr*.tw,kf. (145)
75 or/66-74 [LMICS] (3684327)
76 65 and 75 [UNIQUE PNEUMONIA - LMICS] (673)

****
Embase

Database: Embase <1974 to 2018 August 27>
Search Strategy:
----
1 pneumovirus/ (63)
2 exp human respiratory syncytial virus/ (3056)
3 respiratory syncytial virus infection/ (3982)
4 (respiratorysync#tial virus* or respiratory sync#tial virus* or sync#tial respiratory virus*).tw,kw. (14761)
5 (respiratorysync#tial pneumovirus* or respiratory sync#tial pneumovirus* or sync#tial respiratory pneumovirus*).tw,kw. (0)
6 (HRSV or RSV).tw,kw. (14309)
7 RS virus*.tw,kw. (478)
8 sync#tial virus*.tw,kw. (14814)
9 respiratory syncytial virus vaccine/ (1262)
10 anti-RSV.tw,kw. (382)
11 motavizumab/ (238)
12 motavizumab.tw,kw. (63)
13 ("medi 524" or medi524 or numax).tw,kw. (88)
14 palivizumab/ (2613)
15 (palivizumab or abbosynagis or "MEDI 493" or MEDI493 or synagis or synagys).tw,kw. (1864)
16 or/1-15 [RSV EXPOSURE] (22643)
17 respiratory tract infection/ (51475)
18 lower respiratory tract infection/ (10287)
19 respiratory tract disease/ (51224)
20 (airway? adj2 infect*).tw,kw. (3775)
21 (respiratory tract? adj2 infect*).tw,kw. (29588)
22 (LRTI or LRTIs).tw,kw. (1776)
23 (LRI or LRIs).tw,kw. (642)
24 (respiratory adj (illness* or infect*)).tw,kw. (34223)
25 exp bronchiolitis/ (19375)
26 bronchiolitis.tw,kw. (15344)
27 ((airflow* or air flow* or lung function*) adj2 defici*).tw,kw. (214)
28 or/17-27 [RESPIRATORY INFECTIONS - GENERAL] (157834)
29 16 or 28 [RSV, RESPIRATORY INFECTIONS] (171061)
30 exp child/ (2329953)
31 (infant or infants or infanc* or baby or babies or boy or boys or child* or girl or girls or preschool* or pre-school* or toddler*).tw,kw. (1987382)
32 exp Pediatrics/ (93738)
33 (pediatric* or paediatric*).tw,kw. (479573)
34 (newborn* or neonat* or premie?).tw,kw. (432377)

35 (very low birth weight* or VLBW or VLBWs or "small for gestational age" or "small for gestational ages" or SGA or SGAs).tw,kw. (26748)
36 or/30-35 [INFANT/CHILD POPULATION] (3136278)
37 29 and 36 (58296)
38 exp asthma/ (231534)
39 asthma*.tw,kw. (204655)
40 (bronchospasm* or broncho-spasm* or bronchial spasm*).tw,kw. (7275)
41 abnormal respiratory sound/ (7153)
42 wheezing/ (23610)
43 wheez*.tw,kw. (20096)
44 bronchus hyperreactivity/ (12081)
45 respiratory tract allergy/ (8789)
46 ((respirat* or airway or airways or lung or lungs or bronchi* or broncho* or bronchu*) adj3 (sensitivit* or hypersensitivit* or hyper-sensitivit* or hyperreactiv* or hyper-reactiv*)).tw,kw. (11155)
47 hypersensitivity/ (28283)
48 (allergy or allergies or allergic*).tw,kw. (212257)
49 lung injury/ (29919)
50 ((lung or lungs) adj3 injur*).tw,kw. (40772)
51 ((airway or airways or lung or lungs or respirat*) adj5 (patholog* or histopatholog* or histo-patholog*)).tw,kw. (22853)
52 or/38-51 [ASTHMA/WHEEZE/LRTIs] (519638)
53 37 and 52 [RSV - INFANT/CHILD - ASTHMA/WHEEZE/LRTIs] (14105)
54 exp animal/ or exp animal experimentation/ or exp animal model/ or exp animal experiment/ or nonhuman/ or exp vertebrate/ (24661828)
55 exp human/ or exp human experimentation/ or exp human experiment/ (18686881)
56 54 not 55 (5975858)
57 53 not 56 [ANIMAL-ONLY REMOVED] (13643)
58 editorial.pt. (572532)
59 letter.pt. not (letter.pt. and randomized controlled trial/) (1021368)
60 57 not (58 or 59) [OPINION PIECES REMOVED] (13251)
61 conference abstract.pt. (3126460)
62 60 not 61 [CONFERENCE ABSTRACTS REMOVED] (10627)
63 exp pneumonia/ (257585)
64 pneumoni*.tw,kw. (219841)
65 63 or 64 [PNEUMONIA] (345733)
66 36 and 65 (64938)
67 52 and 66 (8131)
68 67 not 56 (7649)
69 68 not (58 or 59) (7451)
70 69 not 61 (5682)
71 70 not 62 [UNIQUE PNEUMONIA REFS] (3385)
72 Developing Country.sh,kw. (88915)
73 (Africa? or Asia? or Caribbean or West Indies or South America? or Latin America? or Central America?).hw,kw,tw,cp. (578581)
74 (Afghanistan or Albania or Algeria or Angola or Antigua or Barbuda or Argentina or Armenia or Armenian or Aruba or Azerbaijan or Bahrain or Bangladesh or Barbados or Benin or Byelarus or Byelorussian or Belarus or Belorussian or Belorussia or Belize or Bhutan or Bolivia or Bosnia or Herzegovina or Hercegovina or Botswana or Brasil or Brazil or "Brazzavilee" or Bulgaria or Burkina Faso or Burkina Fasso or Upper Volta or Burundi or Urundi or Cambodia or Khmer Republic or Kampuchea or Cameroon or Cameroons or Cameron or Camerons or "Canary Islands" or Cape Verde or Central African Republic or Chad or Chile or China or Colombia or Comoros or Comoro Islands or Comores or Mayotte or Congo or Zaire or Costa Rica or Cote d'Ivoire or Ivory Coast or Croatia or Cuba or Cyprus or Czechoslovakia or Czech Republic or Slovakia or Slovak Republic or Djibouti or French Somaliland or Dominica or Dominican Republic or East Timor or East Timur or Timor Leste or Ecuador or Egypt or United Arab Republic or El Salvador or Eritrea or Estonia or Ethiopia or Fiji or Gabon or Gabonese Republic or Gambia or Gaza or Georgia Republic or Georgian Republic or Ghana or Gold Coast or Greece or Grenada or Guatemala or Guinea or Guam or Guiana or Guyana or Haiti or Honduras or Hungary or India or Maldives or Indonesia or Iran or Iraq or

Isle of Man or Jamaica or Jamahiriya? or Jamahiryia? or Jordan or Kazakhstan or Kazakh or Kenya or Kiribati or Korea or Kosovo or Kyrgyzstan or Kirghizia or Kyrgyz Republic or Kirghiz or Kirgizstan or Lao PDR or Laos or Latvia or Lebanon or Lesotho or Basutoland or Liberia or Libia or Libya or Lithuania or Macedonia or Madagascar or Maghreb or Maghrib or Malagasy Republic or Malaysia or Malaya or Malay or Mayote or Mocambique or Principe or Reunion or Sabah or Sarawak or Malawi or Nyasaland or Mali or Malta or Marshall Islands or Mauritania or Mauritius or Agalega Islands or Mexico or Micronesia or Middle East or Moldova or Moldovia or Moldovian or Mongolia or Montenegro or Morocco or Ifni or Mozambique or Myanmar or Myanma or Burma or Namibia or Nepal or Netherlands Antilles or New Caledonia or Nicaragua or Niger or Nigeria or Northern Mariana Islands or Oman or Muscat or Pakistan or Palau or Palestine or Panama or Paraguay or Peru or Philippines or Philipines or Phillipines or Phillippines or Poland or Portugal or Puerto Rico or Romania or Rumania or Roumania or Russia or Russian or Rwanda or Ruanda or Saint Kitts or St Kitts or Nevis or Saint Lucia or St Lucia or Saint Vincent or St Vincent or Grenadines or Samoa or Samoan Islands or Navigator Island or Navigator Islands or "St Helena" or "Saint Helena" or Sao Tome or Saudi Arabia or Senegal or Serbia or Montenegro or Seychelles or Sierra Leone or Slovenia or Sri Lanka or Ceylon or Solomon Islands or Somalia or South Africa or Sudan or Suriname or Surinam or Swaziland or Syria or Tajikistan or Tadzhikistan or Tadjikistan or Tadzhik or Tanzania or Thailand or Togo or Togolese Republic or Tonga or Trinidad or Tobago or Tunisia or Turkey or Turkmenistan or Turkmen or Uganda or Ukraine or Uruguay or USSR or Soviet Union or Union of Soviet Socialist Republics or Uzbekistan or Uzbek or Vanuatu or New Hebrides or Venezuela or Vietnam or Viet Nam or West Bank or "Western Sahara" or "Western Saharan" or Yemen or Yugoslavia or Zambia or Zimbabwe or Rhodesia).hw,kw,tw,cp. (3627458)
75 ((developing or less* developed or under developed or underdeveloped or middle income or low* income or underserved or under served or deprived or poor*) adj (countr* or nation? or population? or world)).tw,kw. (108129)
76 ((developing or less* developed or under developed or underdeveloped or middle income or low* income) adj (economy or economies)).tw,kw. (559)
77 (low* adj (gdp or gnp or gross domestic or gross national)).tw,kw. (313)
78 (low adj3 middle adj3 countr*).tw,kw. (12778)
79 (lmic or lmics or third world or lami countr*).tw,kw. (6889)
80 transitional countr*.tw,kw. (207)
81 or/72-80 [LMICS] (4029639)
82 71 and 81 [PNEUMONIA - LMICS] (844)
83 conference abstract.pt. (3126460)
84 82 not 83 [CONFERENCE ABSTRACTS REMOVED] (844)
85 69 and 61 [PNEUMONIA - CONFERENCE ABSTRACTS] (1769)
86 85 and 81 [PNEUMONIA - LMICS - CONFERENCE ABSTRACTS] (306)
87 limit 86 to yr="2016-current" (79)
88 82 or 87 [MOST RECENT 2 YRS CONF ABSTRACTS RETAINED] (923)

****

APPENDIX E3. PRISMA CHECKLIST

Section/topic	#	Checklist item	Reported on page #
**TITLE**			
Title	1	Identify the report as a systematic review, meta-analysis, or both.	1
**ABSTRACT**			
Structured summary	2	Provide a structured summary including, as applicable: background; objectives; data sources; study eligibility criteria, participants, and interventions; study appraisal and synthesis methods; results; limitations; conclusions and implications of key findings; systematic review registration number.	2
**INTRODUCTION**			
Rationale	3	Describe the rationale for the review in the context of what is already known.	5
Objectives	4	Provide an explicit statement of questions being addressed with reference to participants, interventions, comparisons, outcomes, and study design (PICOS).	6-7 (Tables 1 & 2)
**METHODS**			
Protocol and registration	5	Indicate if a review protocol exists, if and where it can be accessed (e.g., Web address), and, if available, provide registration information including registration number.	7
Eligibility criteria	6	Specify study characteristics (e.g., PICOS, length of follow-up) and report characteristics (e.g., years considered, language, publication status) used as criteria for eligibility, giving rationale.	7-8 (Table 2)
Information sources	7	Describe all information sources (e.g., databases with dates of coverage, contact with study authors to identify additional studies) in the search and date last searched.	9 (Appendix E2)
Search	8	Present full electronic search strategy for at least one database, including any limits used, such that it could be repeated.	Appendix E2
Study selection	9	State the process for selecting studies (i.e., screening, eligibility, included in systematic review, and, if applicable, included in the meta-analysis).	9-10
Data collection process	10	Describe method of data extraction from reports (e.g., piloted forms, independently, in duplicate) and any processes for obtaining and confirming data from investigators.	9-10

Data items	11	List and define all variables for which data were sought (e.g., PICOS, funding sources) and any assumptions and simplifications made.	9-10 (Table 3)
Risk of bias in individual studies	12	Describe methods used for assessing risk of bias of individual studies (including specification of whether this was done at the study or outcome level), and how this information is to be used in any data synthesis.	11
Summary measures	13	State the principal summary measures (e.g., risk ratio, difference in means).	10
Synthesis of results	14	Describe the methods of handling data and combining results of studies, if done, including measures of consistency (e.g., I^2) for each meta-analysis.	10-11

Table E1. Newcastle-Ottawa Scale rating for risk of bias

Study/Article Name	Outcome	Exposure	Comparator	Outcome Age	Newcastle-Ottawa Ratings[a]		
					Selection	Comparability	Outcome
**Ruotsalainen 2013**							
Backman 2018[66]	Current asthma	RSV-LRTI	Population control group	17-19 yrs.	***	**	***
Ruotsalainen 2013[65]	Current physician-dx. asthma:	RSV-LRTI	Population control group	15-18 yrs.	***	**	*
Ruotsalainen 2013	Current self-reported asthma:	RSV-LRTI	Population control group	15-18 yrs.	***	**	*
**Stein 1999**							
Voraphani 2014[100]	Current asthma	RSV-LRTI	No LRTI	22, 24, 26, and 29 yrs.	****	**	*
Voraphani 2014	Current wheeze	RSV-LRTI	No LRTI	22, 24, 26, and 29 yrs.	****	**	*
Stein 1999[99]	Infrequent wheeze	RSV-LRTI	No LRTI	12-13 yrs.	****	**	*
Stein 1999	Frequent wheeze	RSV-LRTI	No LRTI	5-6 yrs.	****	**	*
Stein 1999	Frequent wheeze	RSV-LRTI	No LRTI	7-8 yrs.	****	**	*
Stein 1999	Frequent wheeze	RSV-LRTI	No LRTI	10-11 yrs.	****	**	*
Stein 1999	Frequent wheeze	RSV-LRTI	No LRTI	12-13 yrs.	****	**	*
Stein 1999	Infrequent wheeze	RSV-LRTI	No LRTI	5-6 yrs.	****	**	*
Stein 1999	Infrequent wheeze	RSV-LRTI	No LRTI	7-8 yrs.	****	**	*
Stein 1999	Infrequent wheeze	RSV-LRTI	No LRTI	10-11 yrs.	****	**	*
**Poorisrisak 2010**							
Poorisrisak 2010[37]	Current asthma	RSV-LRTI hospitalization	No RSV-LRTI hospitalization	$M = 7.6$	***	**	**
**Fjaerli 2005**							
Fjaerli 2005[76]	Physician-managed asthma	RSV-LRTI hospitalization	No respiratory hospitalization	7 yrs.	***	*	**
Fjaerli 2005	Recurrent wheeze	RSV-LRTI hospitalization	No respiratory hospitalization	7 yrs.	***	*	**
**Korppi 1994**							
Korppi 1994[67]	Bronchial asthma	RSV-LRTI hospitalization	Control group from non-atopic and non-asthmatic families	8-9.5 yrs.	**	None	**
Korppi 2004[68]	Current physician-dx. asthma	RSV-LRTI hospitalization	No evidence RSV infection	17-20 yrs.	**	*	**
Korppi 2004	Previous asthma & current wheeze/ prolonged cough	RSV-LRTI hospitalization	No evidence RSV infection	17-20 yrs.	**	*	*
Backman 2014[71]	Physician-dx. asthma	RSV-LRTI hospitalization	Population-based control group	28-31 yrs.	***	None	**

Backman 2014	Self-reported asthma	RSV-LRTI hospitalization	Population-based control group	28-31 yrs.	***	None	*
Ruotsalainen 2010b[70]	Self-reported asthma	RSV-LRTI hospitalization	Population-based control group	25-29 yrs.	***	**	*
Ruotsalainen 2010b	Physician-dx. asthma	RSV-LRTI hospitalization	Population-based control group	25-29 yrs.	***	**	*
Ruotsalainen 2010a[69]	Current physician-dx. asthma	RSV-LRTI hospitalization	Control Group 2: non-selected	~25-27 yrs.	***	*	*
Ruotsalainen 2010a	Current self-reported asthma	RSV-LRTI hospitalization	Control Group 2: non-selected	~25-27 yrs.	***	*	*
Ruotsalainen 2010a		RSV-LRTI hospitalization	Control Group 1: non-atopic and non-asthmatic families	~25-27 yrs.	***	None	*
Ruotsalainen 2010a	Current self-reported asthma	RSV-LRTI hospitalization	Control Group 1: non-atopic and non-asthmatic families	~25-27 yrs.	***	None	*
**Henderson 2005**							
Henderson 2005[77]	Physician-dx. asthma	RSV-LRTI hospitalization	No bronchiolitis hospitalization	91 mos.	***	**	**
Henderson 2005	Parent-reported wheezing	RSV-LRTI hospitalization	No bronchiolitis hospitalization	~30-42 mos.	***	**	**
Henderson 2005	Parent-reported wheezing	RSV-LRTI hospitalization	No bronchiolitis hospitalization	~69-81 mos.	***	**	**
**Drysdale 2015**							
Drysdale 2015[101]	Wheeze	RSV-LRTI	No LRTI in the first 2 years	~ages 1-2	****	None	*
Drysdale 2015	Asthma	RSV-LRTI	No LRTI in the first 2 years	ages 0-2	****	None	None
**Stensballe 2018**							
Stensballe 2018[39]	Wheeze	RSV-LRTI & no prior wheeze	Non-airway hospitalization & no prior wheeze	~3-16 mos. to 21-34 mos.	****	**	*
Stensballe 2018	Wheeze	RSV-LRTI & no prior wheeze	Non-airway hospitalization & no prior wheeze	~18 mos. to 5 yrs.	****	**	*
**Carroll 2017**							
Carroll 2017	Asthma (propensity matched analysis)	> 0% & ≤20% prophylaxis adherence	≥70% RSV immunoprophylaxis adherence	4.5-6 yrs.	***	**	***
Carroll 2017	Asthma (propensity adjusted model)	No immuno-prophylaxis	≥70% RSV immunoprophylaxis adherence	4.5-6 yrs.	***	**	***
**Carbonell-Estrany 2015**							
Carbonell-Estrany 2015	Simple wheezing	RSV-LRTI hospitalization	No RSV-LRTI hospitalization	~5-6 yrs.	***	**	**
Carbonell-Estrany 2015	Recurrent wheezing	RSV-LRTI hospitalization	No RSV-LRTI hospitalization	~5-6 yrs.	***	**	**
Carbonell-Estrany 2015	Severe recurrent wheeze	RSV-LRTI hospitalization	No RSV-LRTI hospitalization	~5-6 yrs.	***	**	**

**Yoshihara 2013**							
Mochizuki 2017	Atopic asthma	No palivizumab receipt	Palivizumab receipt	0-6 yrs.	***	**	**
Mochizuki 2017	Recurrent wheezing	No palivizumab receipt	Palivizumab receipt	0-6 yrs.	***	**	**
Yoshihara 2013	Physician dx recurrent wheezing	No palivizumab receipt	Palivizumab receipt	0-3 yrs.	***	**	***
**Escobar 2013**							
Escobar 2013	Recurrent wheeze	RSV-LRTI (outpatient encounter)	No medically attended RSV-LRTI	4-5 yrs.	***	**	**
Escobar 2013	Recurrent wheeze	RSV-LRTI (outpatient encounter)	No medically attended RSV-LRTI	1-2 yrs.	***	**	**
Escobar 2013	Recurrent wheeze	RSV-LRTI (uncomplicated hospitalization)	No medically attended RSV-LRTI	1-2 yrs.	***	**	**
Escobar 2013	Recurrent wheeze	RSV-LRTI (prolonged hospitalization)	No medically attended RSV-LRTI	1-2 yrs.	***	**	**
Escobar 2013	Recurrent wheeze	RSV-LRTI (outpatient encounter)	No medically attended RSV-LRTI	3-4 yrs.	***	**	**
Escobar 2013	Recurrent wheeze	RSV-LRTI (outpatient encounter)	No medically attended RSV-LRTI	2-3 yrs.	***	**	**
Escobar 2013	Recurrent wheeze	RSV-LRTI (uncomplicated hospitalization)	No medically attended RSV-LRTI	4-5 yrs.	***	**	**
Escobar 2013	Recurrent wheeze	RSV-LRTI (uncomplicated hospitalization)	No medically attended RSV-LRTI	3-4 yrs.	***	**	**
Escobar 2013	Recurrent wheeze	RSV-LRTI (uncomplicated hospitalization)	No medically attended RSV-LRTI	2-3 yrs.	***	**	**
Escobar 2013	Recurrent wheeze	RSV-LRTI (prolonged hospitalization)	No medically attended RSV-LRTI	4-5 yrs.	***	**	**
Escobar 2013	Recurrent wheeze	RSV-LRTI (prolonged hospitalization)	No medically attended RSV-LRTI	3-4 yrs.	***	**	**
Escobar 2013	Recurrent wheeze	RSV-LRTI (prolonged hospitalization)	No medically attended RSV-LRTI	2-3 yrs.	***	**	**
**Kim 2013**							
Kim 2013	Recurrent wheeze	RSV-LRTI hospitalization (placebo group)	No airway infection	~1.5 to 3 yrs.	**	None	*
**Palmer 2011**							
Palmer 2011	Wheeze diagnosis	RSV-LRTI inpatient claims	No LRTI (comparison group)	1-2 yrs.	***	**	**
Palmer 2011	Wheeze diagnosis	RSV-LRTI outpatient claims	No LRTI (comparison group)	1-2 yrs.	***	**	**
Palmer 2011	Asthma diagnosis	RSV-LRTI inpatient claims	No LRTI (comparison group)	1-2 yrs.	***	**	**
Palmer 2011	Asthma diagnosis	RSV-LRTI outpatient claims	No LRTI (comparison group)	1-2 yrs.	***	**	**
**Kusel 2007**							
Kusel 2007	Persistent wheeze	Wheezy RSV-LRTI	No wheezy RSV-LRTI	exposure - age 5	***	**	***
Kusel 2007	Current wheeze	Non-wheezy RSV-LRTI	No non-wheezy RSV-LRTI	4-5 yrs.	***	**	***

Kusel 2007	Current wheeze	Wheezy RSV-LRTI	No wheezy RSV-LRTI	4-5 yrs.	***	**	***
Kusel 2007	Current asthma	Non-wheezy RSV-LRTI	No non-wheezy RSV-LRTI	4-5 yrs.	***	**	***
Kusel 2007	Current asthma	Wheezy RSV-LRTI	No wheezy RSV-LRTI	4-5 yrs.	***	**	***
Kusel 2007	Persistent wheeze	Non-wheezy RSV-LRTI	No non-wheezy RSV-LRTI	exposure - 5 yrs.	***	**	***
Kusel 2012	Current asthma	Wheezy RSV-LRTI	No wheezy RSV-LRTI	9-10 yrs.	***	**	**
Kusel 2012	Current asthma	Febrile RSV-LRTI	No febrile RSV-LRTI	~9-10 yrs.	***	**	**
Kusel 2012	Persistent wheeze	Wheezy RSV-LRTI	No wheezy RSV-LRTI	~0-10 yrs.	***	**	**
Kusel 2012	Persistent wheeze	Febrile RSV-LRTI	No febrile RSV-LRTI	~0-10 yrs.	***	**	**
**Simoes 2007**							
Simoes 2007	Physician diagnosis recurrent wheeze	Combined untreated group	Palivizumab receipt (3+ doses)	~0-3 yrs.	***	**	***
Simoes 2007	Recurrent wheeze not verified by physician	Combined untreated group	Palivizumab receipt (3+ doses)	~0-3 yrs.	***	**	***
**Blanken 2016**							
Blanken 2016	Recurrent wheeze	RSV-LRTI hospitalization	No RSV-LRTI hospitalization	enrollment to 1 yr.	***	**	None
**Bloemers 2010**							
Bloemers 2010	Physician dx. recurrent wheeze	RSV-LRTI (with and without Down Syndrome)	No RSV-LRTI	~0-3 yrs.	**	None	**
Bloemers 2010	Parent-reported recurrent wheeze	RSV-LRTI (with and without Down Syndrome)	No RSV-LRTI	~0-3 yrs.	**	None	*
Bloemers 2010	Physician-dx. asthma	RSV-LRTI (with and without Down Syndrome)	No RSV-LRTI	~0-3 yrs.	**	None	*
**Garcia-Garcia 2007**							
Garcia-Garcia 2007	Asthma	RSV-LRTI hospitalization	Non-LRTI hospitalization	approx. age up to 5	***	**	*
Garcia-Garcia 2007	Recurrent wheeze	RSV-LRTI hospitalization	Non-LRTI hospitalization	~2-3 yrs.	***	None	**
Garcia-Garcia 2007	Recurrent wheeze	RSV-LRTI hospitalization	Non-LRTI hospitalization	~4-5 yrs.	***	None	**
**Mikalsen 2012**							
Mikalsen 2012	Current asthma	RSV-LRTI hospitalization	No RSV-LRTI hospitalization	~10-13 mos. to ~0-3 yrs.	***	None	**
**Osundwa 1993**							
Osundwa 1993	Recurrent wheeze	RSV-LRTI hospitalization	Non-LRTI hospitalization	~3-8 mos. to 2-3 yrs.	***	*	**
**Palmer 2010**							
Palmer 2010	Infantile asthma	RSV-LRTI diagnosis	No LRTI diagnosis	~0-2 yrs.	***	None	*
Palmer 2010	Recurrent wheeze	RSV-LRTI diagnosis	No LRTI diagnosis	~0-2 yrs.	***	None	*

Palmer 2010	Infantile asthma	RSV-LRTI hospitalization	No LRTI diagnosis	~0-2 yrs.	***	None	*
**Weber 1999**							
Weber 1999	Episodes of ALRI with wheezing	RSV-LRTI	Combined control group	0-3 yrs.	***	**	*
**Prais 2016**							
Prais 2016	Parent report of child asthma dx.	No palivizumab	Palivizumab receipt	~0-2 yrs.	***	*	None
Prais 2016	Wheezing episodes	No palivizumab	Palivizumab receipt	~0-2 yrs.	***	*	
Prais 2016	Wheezing episodes	No palivizumab	Palivizumab receipt	~8-9 yrs.	***	*	None
**Fauroux 2014**							
Fauroux 2014	Wheezing	RSV-LRTI hospitalization	No RSV-LRTI hospitalization	~0-2 yrs.	***	**	*
Fauroux 2014	Recurrent wheeze	RSV-LRTI hospitalization	No RSV-LRTI hospitalization	~0-2 yrs.	***	None	*
**Sigurs 1995**							
Sigurs 1995	Recurrent wheeze	RSV-LRTI hospitalization	No respiratory hospitalizations	exposure to age 3	***	**	***
Sigurs 1995	Any wheeze	RSV-LRTI hospitalization	No respiratory hospitalizations	exposure to age 1	***	**	***
Sigurs 1995	Any wheeze	RSV-LRTI hospitalization	No respiratory hospitalizations	exposure to age 3	***	**	***
Sigurs 1995	Asthma	RSV-LRTI hospitalization	No respiratory hospitalizations	exposure to age 1	***	**	**
Sigurs 1995	Asthma	RSV-LRTI hospitalization	No respiratory hospitalizations	exposure to age 3	***	**	**
Sigurs 1995	Recurrent wheeze	RSV-LRTI hospitalization	No respiratory hospitalizations	exposure to age 1	***	**	***
Sigurs 2010	Current asthma or recurrent wheeze	RSV-LRTI hospitalization	No respiratory hospitalizations	18 yrs.	***	**	**
Sigurs 2005	Current asthma or recurrent wheeze	RSV-LRTI hospitalization	No RSV-LRTI hospitalization	0-13 yrs.	***	**	***
Sigurs 2000	Asthma	RSV-LRTI hospitalization	No RSV-LRTI hospitalization	~1-7.5 yrs.	***	**	***
Sigurs 2000	Any wheezing (cumulative)	RSV-LRTI hospitalization	No RSV-LRTI hospitalization	~1-7.5 yrs.	***	**	***
**Zomer-Kooijker 2014**							
Zomer-Kooijker 2014	Current wheeze	RSV-LRTI hospitalization	No RSV-LRTI hospitalization	5-6 yrs.	***	**	*
Zomer-Kooijker 2014	Current parent-reported asthma diagnosis	RSV-LRTI hospitalization	No RSV-LRTI hospitalization	5-6 yrs.	***	**	*
Zomer-Kooijker 2014	Parent-reported ever physician asthma diagnosis	RSV-LRTI hospitalization	No RSV-LRTI hospitalization	5-6 yrs.	***	**	*
**Sims 1978**							
Sims 1978	Wheezing	RSV-LRTI hospitalization	No RSV-LRTI hospitalization	0-1 to 8 yrs.	***	*	*

**Pullan 1982**							
Pullan 1982	History of wheeze	RSV-LRTI hospitalization	No RSV-LRTI hospitalization	0-1 to ~10 yrs.	***	*	*
**Haerskjold 2017**							
Haerskjold 2017	Asthma: hospital contacts/ prescriptions (propensity score model)	No palivizumab receipt	Palivizumab receipt	0-4 yrs.	***	**	**
Haerskjold 2017	Asthma: hospital contacts (propensity score model)	No palivizumab receipt	Palivizumab receipt	0-4 yrs.	***	**	**
Haerskjold 2017	Asthma: hospital contacts/ prescriptions (Cox model)	No palivizumab receipt	Palivizumab receipt	0-4 yrs.	***	**	**
Haerskjold 2017	Asthma: hospital contacts (Cox model)	No palivizumab receipt	Palivizumab receipt	0-4 yrs.	***	**	**
**Juntti 2003**							
Juntti 2003	Asthma ever	RSV-LRTI hospitalization	No RSV-LRTI hospitalization	6-10 yrs.	**	**	*
Juntti 2003	Wheezing ever	RSV-LRTI hospitalization	No RSV-LRTI hospitalization	6-10 yrs.	**	**	*
Juntti 2003	Wheezing in the past year	RSV-LRTI hospitalization	No RSV-LRTI hospitalization	6-10 yrs.	**	**	*
**Bertrand 2015**							
Bertrand 2015	Asthma diagnosis	RSV-LRTI hospitalization	Controls undergoing a procedure for non-infectious disease	0-1 yrs.to 3-4 yrs.	**	None	*
Bertrand 2015	Recurrent wheeze	RSV-LRTI hospitalization	Controls undergoing a procedure for non-infectious disease	0-1 yrs.to 3-4 yrs.	**	None	*
**Singleton 2003**							
Singleton 2003	Diagnosis of reactive airway disease or asthma	RSV-LRTI hospitalization	No RSV-LRTI hospitalization	2-6 yrs.	***	*	**
Singleton 2003	Current wheezing (adjusted for breastfeeding)	RSV-LRTI hospitalization	No RSV-LRTI hospitalization	5-8 yrs.	***	**	**
Singleton 2003	Current wheezing (adjusted for smoking)	RSV-LRTI hospitalization	No RSV-LRTI hospitalization	5-8 yrs.	***	**	**
Singleton 2003	Current wheezing (adj. for prematurity)	RSV-LRTI hospitalization	No RSV-LRTI hospitalization	5-8 yrs.	***	**	**
**Schauer 2002**							
Schauer 2002	Recurrent wheezing	RSV-LRTI hospitalization	No RSV-LRTI hospitalization	0-1 yrs. to 6-18 mos.	***	**	*
Schauer 2002	Wheezing	RSV-LRTI hospitalization	No RSV-LRTI hospitalization	0-1 yrs. to 6-18 mos.	***	**	*
**Caliskan 2013b (COPSAC cohort)**							
Bonnelykke 2015	Physician dx. asthma (adj.for # respiratory episodes)	RSV-LRTI	No RSV$^+$ samples via viral surveillance	0-7 yrs.	****	*	*
Bonnelykke 2015	Physician dx. asthma (not adj.for # respiratory episodes)	RSV-LRTI (not adjusted for # of respiratory episodes)	No RSV$^+$ samples via viral surveillance	0-7 yrs.	****	None	*

Caliskan 2013b	Physician dx. asthma	RSV-LRTI (homozygous non-risk genotype)	No RSV^{+} samples via viral surveillance (homozygous non-risk genotype)	0-7 yrs.	****	**	*
**dos Santos Simoes 2018**							
dos Santos Simoes 2018	Recurrent wheeze	No palivizumab receipt	Palivizumab receipt	prophylaxis to age 18-54 months	**	None	None
**Stensballe 2009**							
Stensballe 2009	Asthma hospitalization	RSV-LRTI hospitalization	No RSV hospitalization	exposure (0-5 yrs.) to 3-5 mos. later	***	**	*
Stensballe 2009	Asthma hospitalization	RSV-LRTI hospitalization	No RSV hospitalization	exposure (0-5 yrs.) to 6-11 mos. later	***	**	*
Stensballe 2009	Asthma hospitalization	RSV-LRTI hospitalization	No RSV hospitalization	exposure (0-5 yrs.) to 1-2 yrs. later	***	**	**
Stensballe 2009	Asthma: inhaled corticosteroids 2+ times in 12 months	RSV-LRTI hospitalization	No RSV hospitalization	exposure (0-5 yrs.) to 3-5 mos. later	***	**	*
Stensballe 2009	Asthma: defined as use of inhaled corticosteroids	RSV-LRTI hospitalization	No RSV hospitalization	exposure (0-5 yrs.) to 6-11 mos. later	***	**	*
Stensballe 2009	Asthma: defined as use of inhaled corticosteroids	RSV-LRTI hospitalization	No RSV hospitalization	exposure (0-5 yrs.) to 1-2 yrs. later	***	**	**
Stensballe 2009	Asthma: defined as use of inhaled corticosteroids	RSV-LRTI hospitalization	No RSV hospitalization	exposure: 0-5 years old; outcome: >2 years after RSV hospitalization ages 0-5	***	**	**
**Broughton 2007**							
Broughton 2007	Wheezing	RSV-LRTI	No RSV^{+} samples via viral surveillance	11-12 mos. corrected for gestational age at birth	****	None	*
**Munywoki 2013**							
Munywoki 2013	Pneumonia with wheeze	RSV-LRTI hospitalization	Non-LRTI hospitalization	exposure (0-12 months) to 5 yrs.	***	**	**
**Lemanske 2005**							
Rubner 2017	Current asthma	RSV-LRTI	No RSV-LRTI	12-13 yrs.	****	**	**
Rubner 2017	Current asthma	RSV-LRTI	No RSV-LRTI	10-11 yrs.	****	**	**
Rubner 2017	Current asthma	RSV-LRTI	No RSV-LRTI	7-8 yrs.	****	**	**
Rubner 2017	Current asthma	RSV-LRTI	No RSV-LRTI	5-6 yrs.	****	**	**
Caliskan 2013a	Asthma diagnosis	RSV-LRTI (homozygous non-risk genotype)	No RSV-LRTI (homozygous non-risk genotype)	6-8 yrs.	****	**	*
Lemanske 2005	Wheezing respiratory illness	RSV-LRTI moderate-severe illness without wheeze	No RSV-LRTI	~2-3 yrs.	****	**	**
Lemanske 2005	Wheezing respiratory illness	RSV-LRTI moderate-severe illness without wheeze	No RSV-LRTI	~2-3 yrs.	****	**	**

Jackson 2008	Current asthma	RSV-LRTI	No RSV^+ or HRV^+ LRTI	5-6 yrs.	****	*	**
Jackson 2008	Current asthma	RSV-LRTI (no co-infection)	No RSV-LRTI	~5-6 yrs.	****	*	**
Jackson 2008	Current asthma	RSV-LRTI (no co-infection)	No RSV-LRTI	~5-6 yrs.	****	*	**
Jackson 2008	Current asthma	RSV-LRTI	No RSV^+ or HRV^+ LRTI	~5-6 yrs.	****	*	**

[a] Selection (range: 0 to 4 stars), Comparability (range: 0 to 2 stars), Outcome/Exposure (range: 0 to 3 stars); maximum possible score = 9 stars. The Newcastle-Ottawa instrument and manual are available online at: http://www.ohri.ca/programs/clinical_epidemiology/oxford.asp

Table E2. Cochrane Risk of Bias ratings for randomized controlled trials

			Cochrane Risk of Bias Ratings Categories						
Outcome	***Age at Outcome***		***Random sequence generation***	***Allocation concealment***	***Blinding of participants and personnel***	***Blinding (outcome assessment)***	***Incomplete outcome data***[a]	***Selective reporting***	***Other sources of bias.***
**Blanken 2013; Scheltema 2018**									
Parent-reported recurrent wheeze	1		**Low:** Permuted-block randomization stratified by gestational age	**Low:** Intervention assignment unlikely to have been foreseen	**Low:** Double-blinded; research nurses aware of treatment assignment but trained not to reveal	**Low**: Assessors blinded	**Low:** Estimate based on full ITT[b] sample	**Low:** Report on outcomes described in study protocol	**Low:** No other major biases detected
Any parent-reported wheeze	1				**Low:** Double-blinded; research nurses aware of treatment assignment but trained not to reveal		**Low**: Estimate based on full ITT sample		
Physician diagnosed asthma	6				**High:** Assessors blinded but not participants		**High:** Estimate[c] based on n=367 of the N=429 ITT sample (14% missing)		
Parent-reported asthma	6				**High:** Assessors blinded but not participants		**Low:** n=367 of the N=429 ITT sample provide data (14% missing); estimate based on multiple imputation analysis		
Any parent-reported wheeze	1 to 3				**High:** Assessors blinded but not participants		**High:** Estimate[c] based on n=370 of the N=429 ITT sample (14% missing)		
**O'Brien 2015**									
Serious early childhood wheeze	1 to 3		**Low:** Block randomization stratified by site	**Low:** Intervention assignment unlikely to have been foreseen	**Low:** All study participants, investigators, funding staff, and monitoring staff were masked.	**Low**: Assessors blinded	**High:** Estimate based on n=1919 of the N=2127 ITT sample (10% missing)	**Low:** Report on outcomes described in study protocol	**Low:** No other major biases detected
3+ medically-attended wheeze episodes	1 to 3				**Low:** All study participants, investigators, funding staff, and monitoring staff were masked.				
1+ medically-attended wheeze episodes	1 to 3				**Low:** All study participants, investigators, funding staff, and monitoring staff were masked.				

[a]Estimates based on analyses with > 5% of participant data missing were considered high risk of bias unless some effort was made to

[b]ITT=Intention-to-treat sample

[c]Investigators conducted a secondary analysis using multiple imputation to reduce bias due to missing data that produced similar results; however, the multiple-imputation-based estimate was not reported so we used the primary effect estimate, which included only participants who provided outcome data

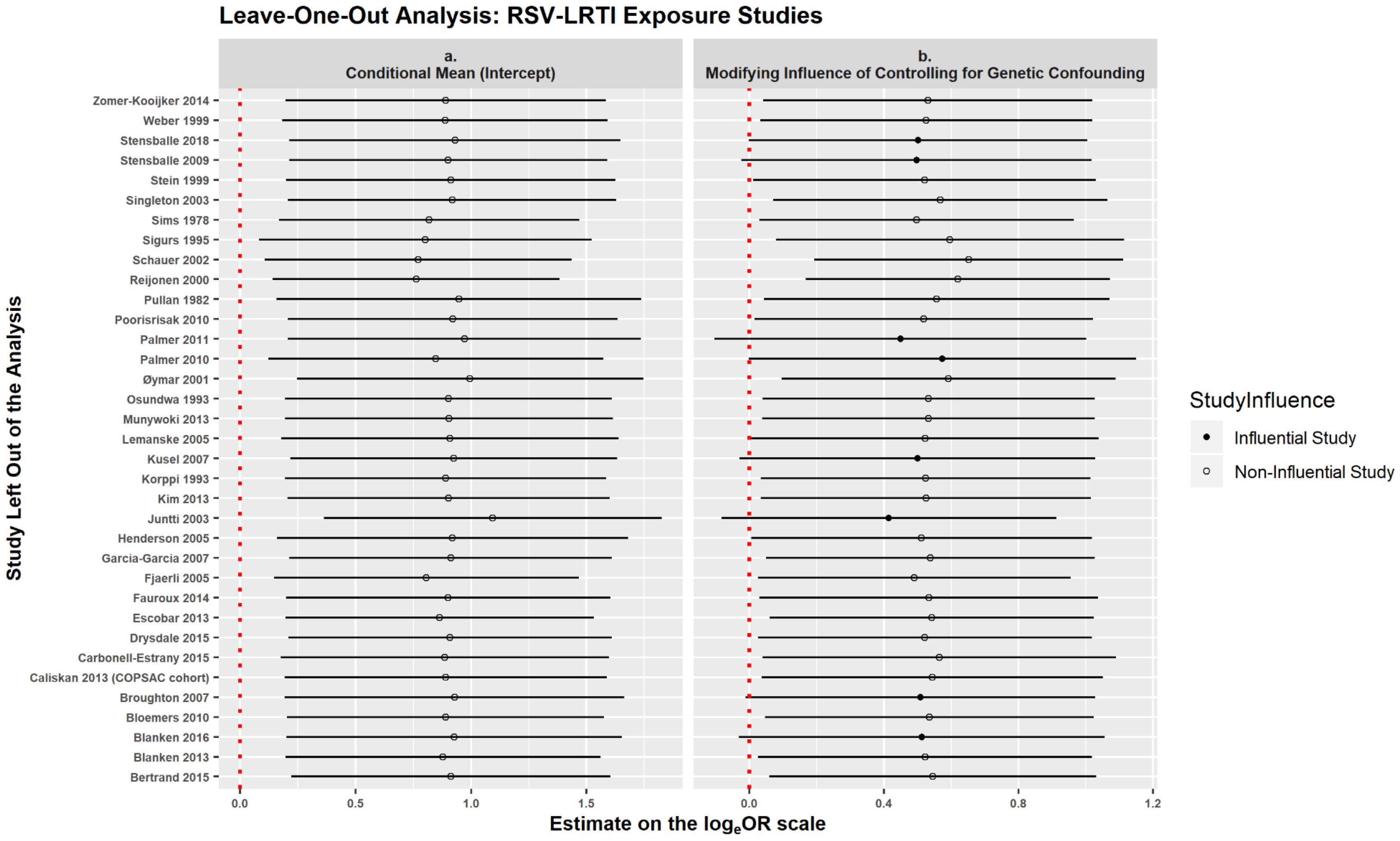

Figure E1. Leave-one-out sensitivity analysis for RSV-LRTI exposure studies. The x-axis estimates from the primary meta-regression model estimating the weighted mean natural log of the odds ratio ($\log_e OR_+$) for the association between RSV-LRTI and subsequent wheezing illness. Each dot represents the mean estimate with the corresponding study listed on y-axis (including all its estimates) removed from the analyses. The horizontal lines going through the points are 95% confidence intervals for the point estimates minus the corresponding study listed on the y-axis. The vertical dotted red lines represent $\log_e OR_+$ values of 0 (i.e., a null effect). For the Intercept estimate (representing the conditional mean $\log_e$OR with all effect modifiers held at their most common levels), no individual

study had sufficient influence to change whether or not the 95% confidence interval around the $\log_e$OR contained the null. The same was not true for the effect modifier coding whether or not studies controlled for genetic confounding. Eight studies had sufficient influence to alter whether the confidence interval around the estimate contained the null value. Influential studies are denoted with filled circles whereas non-influential studies are denoted by open circles.
